# Evaluation of Clove Phytochemicals as Potential Antiviral Drug Candidates Targeting SARS-CoV-2 Main Protease: Computational Docking, Molecular Dynamics Simulation, and Pharmacokinetic Profiling

**DOI:** 10.3389/fmolb.2022.918101

**Published:** 2022-06-28

**Authors:** Arun Chandra Manivannan, Arunkumar Malaisamy, Murugesh Eswaran, Arun Meyyazhagan, Vijaya Anand Arumugam, Kannan R. R. Rengasamy, Balamuralikrishnan Balasubramanian, Wen-Chao Liu

**Affiliations:** ^1^ Department of Microbiology, Alagappa University, Karaikudi, India; ^2^ Integrative Biology Division, International Centre for Genetic Engineering and Biotechnology, New Delhi, India; ^3^ Department of Bioinformatics, Bharathiar University, Coimbatore, India; ^4^ Department of Life Sciences, CHRIST (Deemed to be University), Bengaluru, India; ^5^ Department of Human Genetics and Molecular Biology, Bharathiar University, Coimbatore, India; ^6^ Department of Pharmacology, Centre for Transdisciplinary Research, Saveetha Dental College, Saveetha Institute of Medical and Technical Sciences (SIMATS), Chennai, India; ^7^ Department of Food Science and Biotechnology, College of Life Science, Sejong University, Seoul, South Korea; ^8^ Department of Animal Science, College of Coastal Agricultural Sciences, Guangdong Ocean University, Zhanjiang, China

**Keywords:** clove, casuarictin, phytochemicals, SARS-CoV-2, main protease

## Abstract

The severe acute respiratory syndrome coronavirus 2 (SARS-CoV-2) virus can cause a sudden respiratory disease spreading with a high mortality rate arising with unknown mechanisms. Still, there is no proper treatment available to overcome the disease, which urges the research community and pharmaceutical industries to screen a novel therapeutic intervention to combat the current pandemic. This current study exploits the natural phytochemicals obtained from clove, a traditional natural therapeutic that comprises important bioactive compounds used for targeting the main protease of SARS-CoV-2. As a result, inhibition of viral replication effectively procures by targeting the main protease, which is responsible for the viral replication inside the host. Pharmacokinetic studies were evaluated for the property of drug likeliness. A total of 53 bioactives were subjected to the study, and four among them, namely, eugenie, syzyginin B, eugenol, and casuarictin, showed potential binding properties against the target SARS-CoV-2 main protease. The resultant best bioactive was compared with the commercially available standard drugs. Furthermore, validation of respective compounds with a comprehensive molecular dynamics simulation was performed using Schrödinger software. To further validate the bioactive phytochemicals and delimit the screening process of potential drugs against coronavirus disease 2019, *in vitro* and *in vivo* clinical studies are needed to prove their efficacy.

## Graphical Abstract

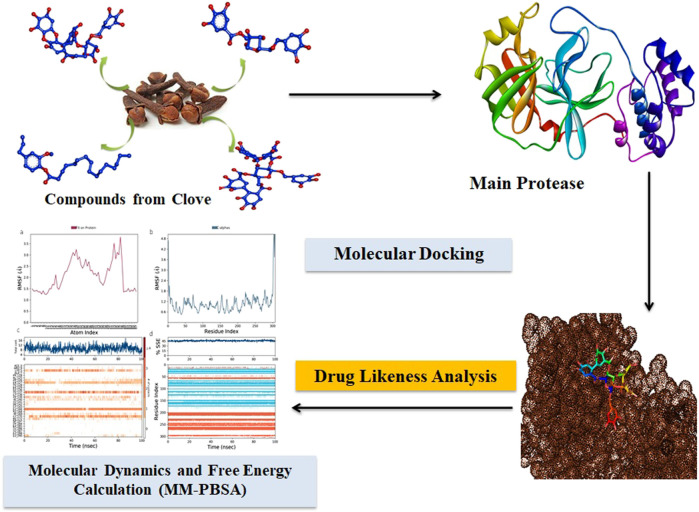

## Introduction

A sudden outbreak of respiratory illness with unknown etiology arose in Wuhan, China, and was later diagnosed as a novel coronavirus (nCoV) with a novel variant called severe acute respiratory syndrome (SARS)-CoV-2. Coronaviruses (CoVs) are a family of enveloped RNA viruses comprising seven human coronaviruses (HCoVs) causing human and animal infection (Biswaranjan, 2022). These are zoonotic obligate intracellular organisms and primarily infect respiratory and associated regions, and this novel virus spreads through air transmission when an infected person coughs or sneezes (Mittal et al., 2020; Majdi et al., 2022). HCoV-229E, HCoV-NL63, HCoV-OC43, and HCoV-HKU1 are usually seasonal, causing mild respiratory diseases that are best known for causing common cold, while other variants like CoV229E and OC43 can provoke pneumonia. Since 3 decades of the virus domination from the first spread of severe strains of middle east respiratory syndrome (MERS)-CoV, SARS-CoV-1, and the current SARS-CoV-2, the lesser known virus is stuck on the headlines for its high pathogenicity and high proliferation rate (Liu et al., 2020; Majdi et al., 2022). The viral infection prevails in one’s body with a highly specific recognition between the virus spike proteins through HCoV-specific receptors. Meanwhile, the main protease (M^Pro^) is the vital enzyme for processing viral polyproteins produced after being translated from RNA. This M^Pro^ is structurally present as a homodimer, which is made up of two promoters of three domains, namely I, II, and III, whose amino acid ranges are 8–101, 102–184, and 201–303, respectively, and a long loop (185–200) that connects domains II and III (Kumar et al., 2021; Mengist et al., 2021). Every possible sector has unleashed its potency over research and development to targeting the SARS-CoV-2 virus to wind this raging pandemic (Marcelino et al., 2022). Traditional medicinal practices based on herbs and their extracts are gaining momentum. Their formulations are widely given with supplementary allopathic treatment for the earlier recovery (Ang et al., 2020; Mhya et al., 2021; Vanshylla et al., 2021; Ashande et al., 2022; Nair et al., 2022). Syzygiumaromaticum, a native species of the Maluku Islands in Indonesia, traditionally found its importance as a flavoring additive for food. However, clove finds its use in ayurvedic and Chinese medicine (Cortés-Rojas et al., 2014). These aromatic flower buds are from a tree species of the Myrtaceae family that inhabits tropical climates. The current status of coronavirus disease 2019 (COVID-19) infection on 5 March 2022 crossed 440,807,756 cases with 5,978,096 deaths worldwide, and India’s status was 42,951,556 cases with 514,589 deaths.

This research evaluates the antiviral efficacy of clove-based phytochemicals by computationally using the autodock protocol by targeting the M^Pro^ of the SARS-CoV-2 virus (KuchiBhotla et al., 2021; Marcelino et al., 2022). Moreover, the computational world has witnessed a recent trend of large natural compound data retrieval to target various structures of the SARS-CoV-2 virus to define a proper therapeutic. The extensive pathophysiological mechanisms underlying viral infections as well as the related adverse effects of the currently available conventional medicines necessitate the development of a novel drug discovery process. With the conventional methods, finding a new drug is very challenging, and also, it is the costliest method ever. *In silico* methods were carried out to facilitate the virtual screening of the best drug candidate to overcome the challenging drawbacks. Pharmacological studies were conducted to analyze the bioavailability and dynamics inside the host by profiling the ADMET properties of phytochemicals for the suitable drug candidate through extending absorption, distribution, metabolism, excretion, and toxicity (ADMET) (Kar et al., 2021). The molecular dynamics simulation studies were conducted to dive deep into their extent and efficiency by sustaining the administered system over a defined time of 100 ns of total simulation. Investigation of clove-extracted compounds against a target of the M^Pro^ of the SARS-CoV-2 virus leads to effective therapeutic drugs. Further validation of all our compounds was compared with the commercial antiviral drugs to combat COVID-19.

## Materials and Methods

### Protein Preparation

The M^Pro^ acts as a target of treatment for various viral infectious agents, including SARS CoV-2, MERS-CoV, noroviruses, enteroviruses, and rhinoviruses. The M^Pro^ is a disparate protein homing in the infected individual, and as it catalyzes most maturation cleavage events, this proteolytic compound is an explicit target for effective lead screening (Luan et al., 2020). The X-ray crystal structure of M^Pro^ (PDB ID-6LU7) was retrieved from the RCSB Protein Data Bank Database. The structure was subjected to protein preparation using autodock tools, a graphical user interface program, which was exploited for the preparation, running, and analyzing the docking simulations. Water molecules, ligand groups (inhibitor), and other nonspecific molecules were removed, polar hydrogen was added with the merging nonpolar hydrogens, and partial charges were assigned (Arunkumar et al., 2021). The grids are placed in the region that possesses the nature of an active site since grids direct the ligand toward the binding site (Forli et al., 2016).

### Ligand Preparation

Data on active phytochemicals present in clove were acquired from the curated databases of Indian Medicinal Plants, Phytochemistry, and Therapeutics (IMPPAT) (Mohanraj et al., 2018). These subsequent structures were retrieved from the PubChem repository, and the related structures were retried from the Zinc Database in the output format of the structure data file. These were converted to the PDB format using Open Babel software; energy minimization was carried out using pyrxtool applying the molecular mechanics force field and optimized for further exploitation of the ligand. The complete dataset of phytochemical names and their IDs used in this study is provided in Supplementary Table S1.

### Molecular Docking

The molecular docking was performed in autodock tools with an extensive suite of python molecular viewers. First, the site-specific docking was carried out with the aid of autodock 4.2; during docking, the protein was placed as a rigid molecule and the ligand was flexible (Trott and Olson., 2010). The studies were carried out using the Lamarckian genetic algorithm with the genetic algorithm parameters comprising 2.5 × 10^6^ energy appraisals and a maximum number of 2.7× 10^4^ generations with a mutation rate of 0.02 with a crossover rate of 0.8. Pseudo Solis and Wets parameters for local search were performed and introduced 300 iterations. Finally, 50 independent runs for each compound were placed, with the grid dimension of 76 × 76 × 76 and with a spacing of 0.375Å (Seeliger and de Groot., 2010).

### Pharmacokinetics Evaluation

Target prediction studies compute the probable macromolecular target site of the screened small molecules; this methodology aids in tracing the bioactivity, side effects, and off-targets. In addition, the ADMET analysis divulges the pharmacokinetics that a ligand must boat to establish its function in the administered body (Arunkumar et al., 2022). The top-ranked compound was evaluated for the ADMET analysis using the Qikrop module on Schrödinger’s Maestro platform (Schrödinger Release 2021-2: QikProp, Schrödinger, LLC, New York, NY, 2021).

### Molecular Dynamics Simulation

Because molecules are dynamic in nature, studying their motions at the molecular and atomistic levels is critical to comprehending the crucial physicochemical processes. In all other computational applications, molecular dynamics simulation stands alone as the essential computational technique for capturing the dynamic events of scientific interest. Based on the molecular interaction and binding score of the small molecule against the target molecule, the top-ranked complex molecules were selected for the molecular dynamics simulation studies. First, the complex molecule was preprocessed using the protein preparation wizard module; then the structure was refined by optimizing the hydrogen bond and applying the force field OPLS3e for energy minimization. OPLS3e improves the accuracy of small-molecule conformational propensities, solvation, and protein–ligand binding performance benchmarks (Roos et al., 2019). Furthermore, the complex molecule was solvated using a system builder module to a hydration model (TIP3P) in the 3D orthorhombic box with a buffer distance of 10 Å. Finally, the whole system is designated for the simulation time of 100 ns with 1,000 frame trajectory points under a default NPT ensemble of constant pressure, temperature, and atom number. They were performed using the Desmond module on Schrödinger’s Maestro platform (Schrödinger Release 2021-2: Desmond Molecular Dynamics System, D. E. Shaw Research, New York, NY, 2021. Maestro-Desmond Interoperability Tools, Schrödinger, New York, NY, 2021).

## Results and Discussion

Despite fast-tracking the research of COVID cure, no potential lead molecules that can effectively break the viral proliferation chain within an individual are identified (Anju et al., 2020). Moreover, many studies are performed on phytochemicals from medicinal plants for their efficacy against the current COVID-19 disease. SARS-CoV-2 viruses are also reported to get disseminated into various body organs and contaminate the environment in more than one route (Xu et al., 2016; Deng-hai Zhang et al., 2020; Garg et al., 2020; Biswaranjan, 2022). The discovery of the SARS-CoV-2 M^Pro^ has opened the door for an effective approach to drug discovery that can be enabled *via* a virtual combinational mode employing computational tools (Guo et al., 2021; Marcelino et al., 2022).

### Molecular Docking and Interactions

About 53 phytochemicals extracted from clove were subjected to molecular docking experiments against the main protease; 27 compounds were native to clove, and 23 are chemicals structurally similar to a few native compounds retrieved from the ZINC database. Around 60–90% of clove phytocompositions are eugenol, eugenyl acetate, caryophyllene, and aceteugenol (Xu et al., 2016). The US Food and Drug Administration (FDA) categorized the clove essential oil as generally recognized as safe, and the World Health Organization has drafted the daily intake quantity for cloves as 2.5 mg/kg of an individual (Sink et al., 2007; Kulkarni et al., 2020). The M^Pro^ is responsible for proper viral replication in SARS CoV-2. Hence, any potential leads can effectively inhibit viral replication inside the host system (Mothay and Ramesh, 2020; Narkhede et al., 2020). Thus, any compound manifesting the disarming of the M^Pro^ can be taken for further clinical studies. Still, various leading laboratories in dry and wet labs worldwide are thriving hard to ace the race to screen for highly efficient drugs for the SARS-CoV-2 virus that could effectively treat any variant of the same.

**FIGURE 1:**
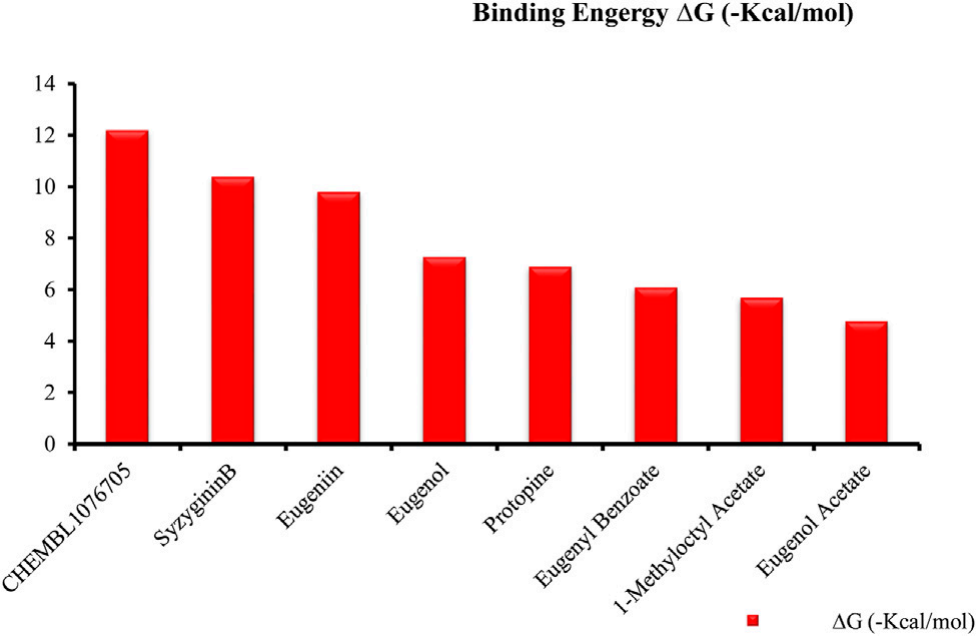
Graphical representation of top-ranked phytochemicals docked against the M^Pro^ unit of binding energy [∆G (−kcal/mol)].

Recently, large quantities of natural compounds are being exploited to act against the deadly virus. A total of 53 compounds were subjected to robust docking using autodock Vina tools against the main viral protease; four compounds, namely, casuarictin, eugeniin, syzyginin B, and eugenol, are identified to bind with the M^Pro^ with the numerically lowest binding energies (most negative) such as −12.2 kcal/mol, −9.8 kcal/mol, −10.4 kcal/mol, and −7.3 kcal/mol, respectively. Since the binding energy score was negative for almost all the compounds, those exhibiting values more than –7 were censored, and the remaining were subjected to further *in silico* modeling (Figure 1).

The complex molecules (protein and ligand) were interrogated for their characters of post docking analysis using the Biovia Discovery studio tool. Hydrogen interaction was the predominant force for bond formation and spatial arrangement of the ligands within interacting pockets. In contrast, hydrophobic and electrostatic interactions are facilitated by a hydrogen bond. Casuarictin demonstrates the highest binding energy with an alliance of five hydrogen bonds with amino acids Thr199 (3.02 Å), Asp197 (3.79 Å), Arg131 (2.76 Å), Lys137 (2.46 Å), and Leu287 (3.53 Å) and three hydrophobic interactions with amino acids Leu287 (5.30Å), Leu272 (5.12 Å), and Tyr239 (5.20 Å).

**FIGURE 2:**
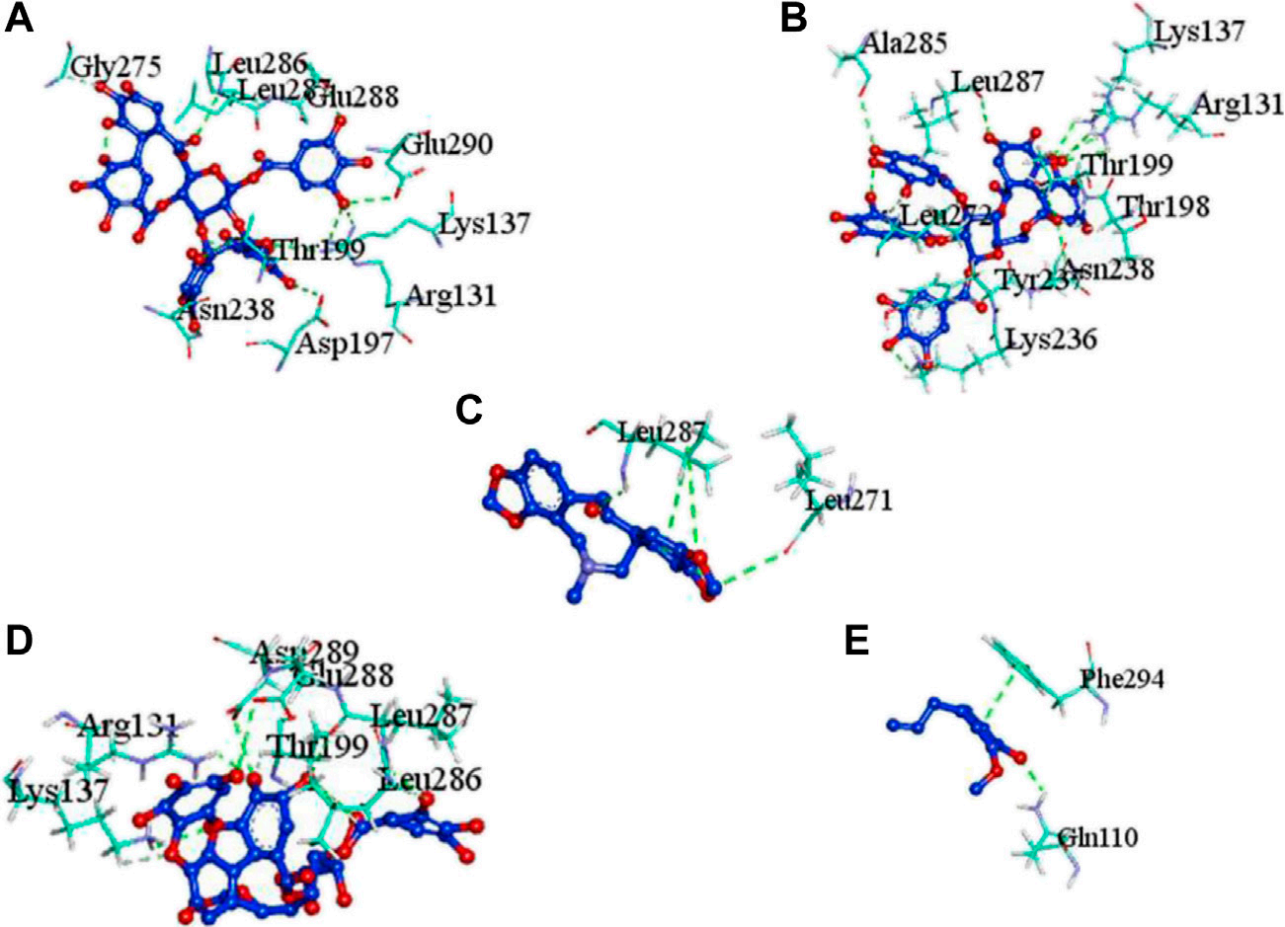
Molecular docking interaction of target proteins with clove’s phytochemicals of **(A)** casuarictin, **(B)** eugeniin, **(C)** protopine, **(D)** syzyginin B, and **(E)** eugenol.

**FIGURE 3:**
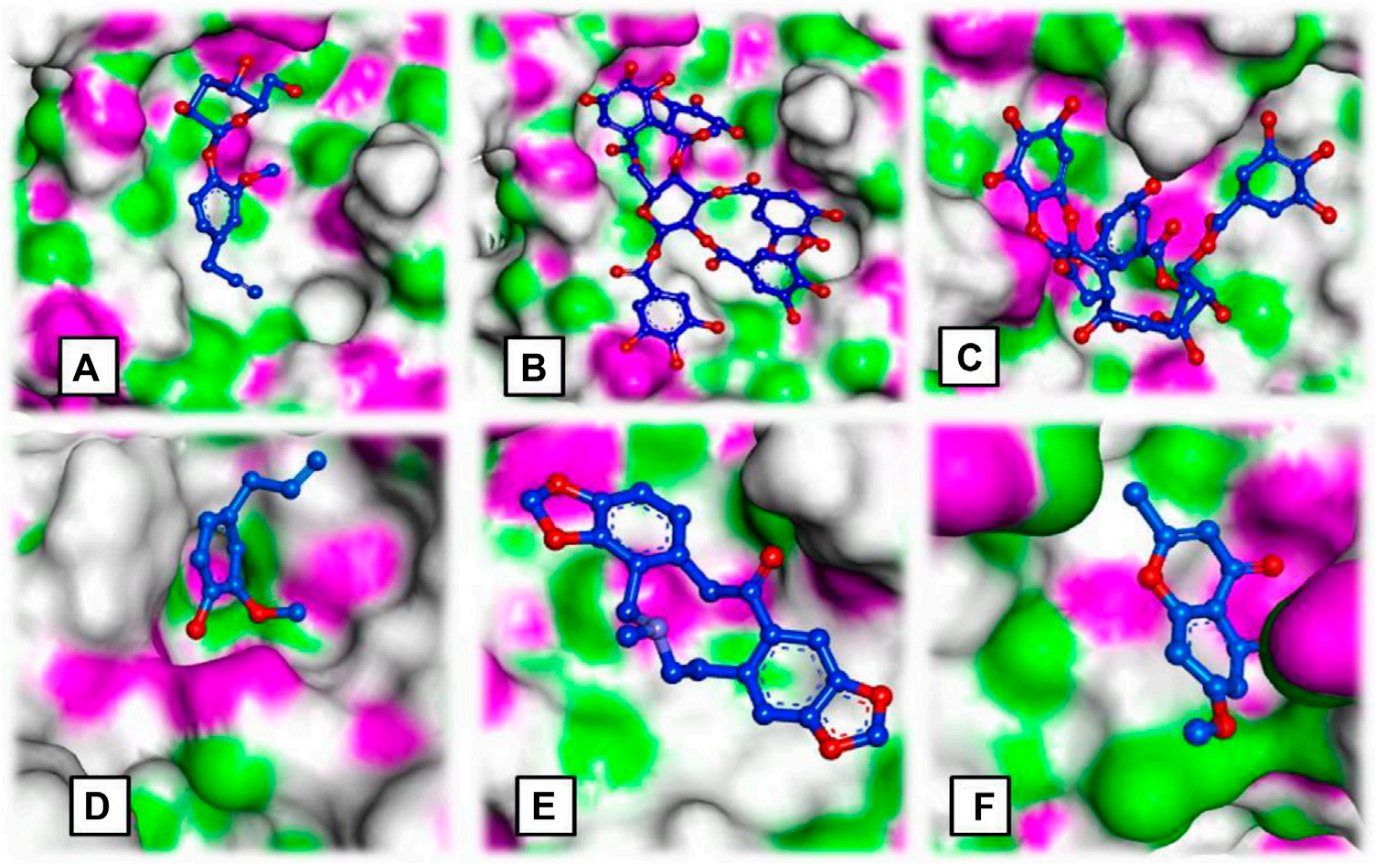
Ligand occupancy in an active site of a target protein complex molecule of **(A)** syringin, **(B)** eugeniin, **(C)** syzyginin, **(B,D)** eugenol acetate, **(E)** protopine, and **(F)** 1-methyloctyl acetate.

**FIGURE 4:**
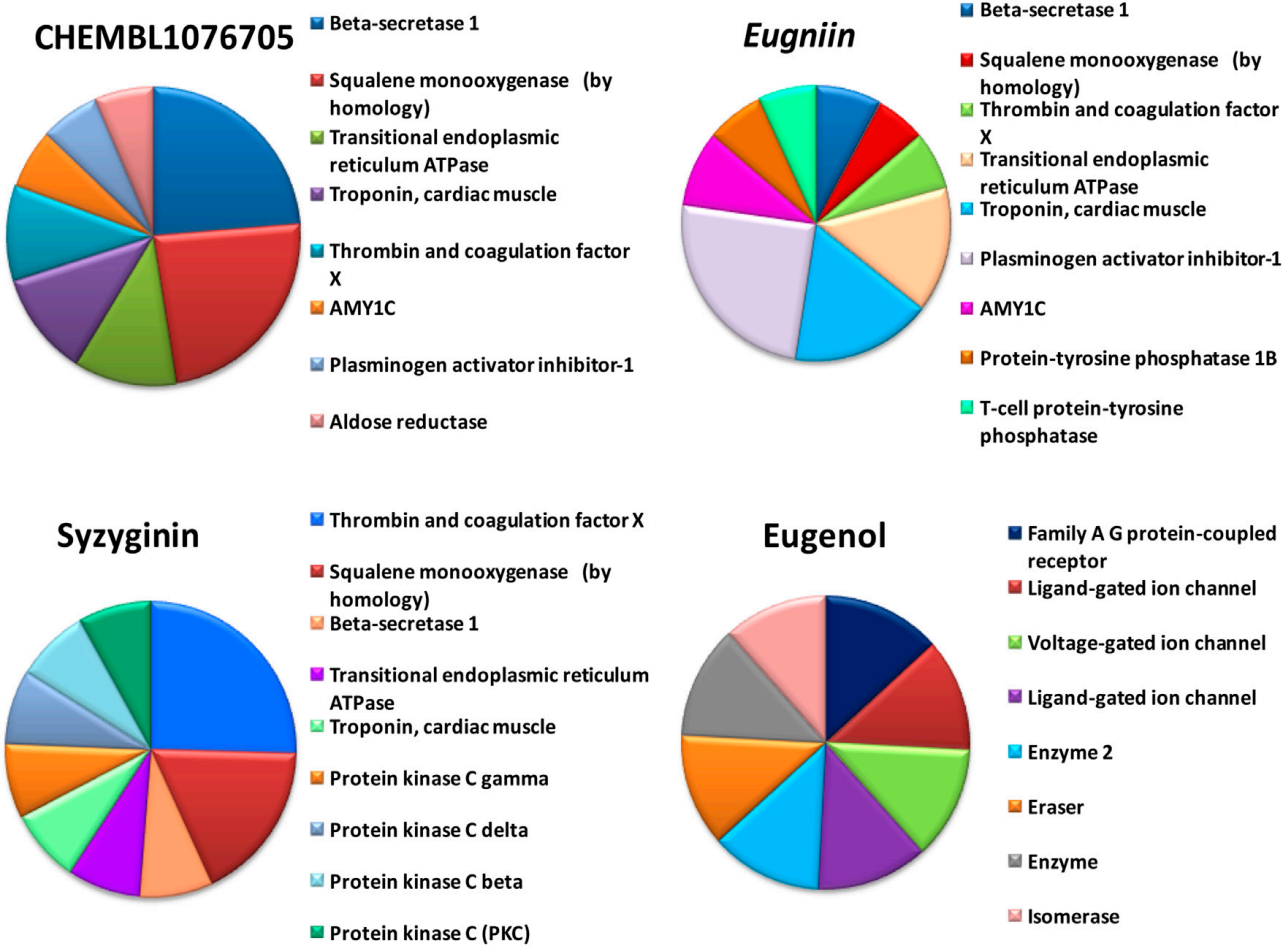
Off-target prediction of selected compounds including beta secretase, squalene mono-oxygenase, thrombin, and coagulation factor.

Next to this, syzyginin B establishes a hydrogen bond with six amino acids, Lys137 (2.70 Å), Arg131 (2.10 Å), Asp289(2.81 Å), Glu228 (3.38 Å), Leu287 (3.33 Å), and Thr199 (2.53 Å), and one amino acid forging the hydrophobic interaction at Leu287 (4.89 Å). Finally, eugeniin with seven hydrogen bonds at locations Ala285 (3.38 Å), Leu287 (3.08 Å), Lys236 (2.67 Å), Asn238(2.57 Å), Lys137 (2.85 Å), Thr199 (3.07 Å), and Arg131 (2.98 Å) fashions a binding energy within the range of −9.8 kcal/mol. Table 1 describes the types of interaction various ligands are experiencing. These bonds are responsible for arresting the ligand efficiently within the active site of the protein. Figures 2, 3, 4 render the delineation of various bonds formed in screened compounds. The remaining 53 complex molecule interactions are listed in Supplementary Table S1, and for the remaining phytochemicals, interactions of their 3D structure with 2D interactions are represented in Supplementary Figures S1–19.

**TABLE 1:** Interaction between the amino acid residue of COVID -19 main protease and ligands at receptor sites.

Ligands	Interacting Species and Bond Distance		
	Hydrogen Binding Interaction	Hydrophobic Interaction	Electrostatic Interaction
Casuarictin, (CHEMBL1076705) 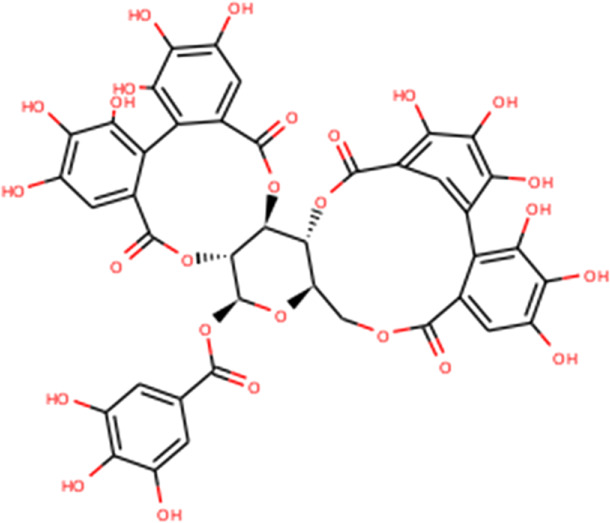	Thr199 (3.02), Asp197 (3.79), Arg131 (2.76), Lys137 (2.46), and Leu287 (3.53)	Leu287 (5.30), Leu272 (5.12), and Tyr239 (5.20)	—
Eugeniin 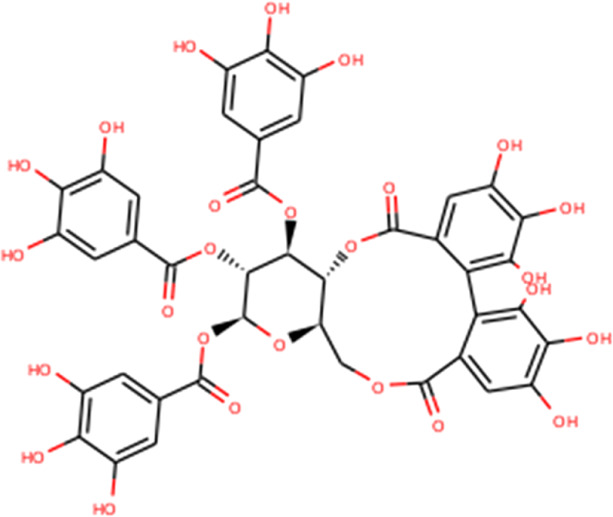	Ala285 (3.38), Leu287 (3.08), Lys236 (2.67), Asn238(2.57), Lys137 (2.85), Thr199 (3.07), and Arg131 (2.98)	Tyr237 (5.04)	Lys236 (3.98)
Syzyginin B 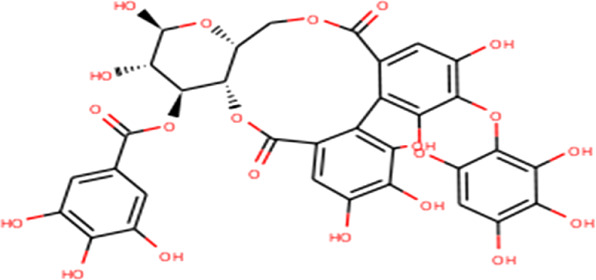	Lys137 (2.70), Arg131 (2.10), Asp289(281), Glu228 (3.38), Leu287 (3.33), and Thr199 (2.53)	Leu287 (4.89)	—
Eugenol 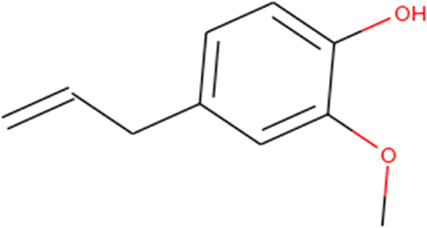	Phe294(3.79), Gln110 (2.29), and Ala129 (1.96)	-	—
Protopine 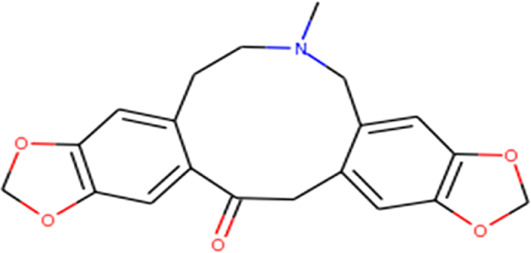	Leu271 (3.46) and Leu287 (2.51)	Leu287 (4.66)	—
Eugenyl Benzoate 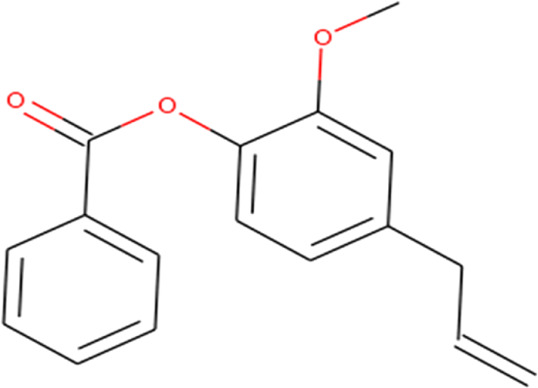	Cys145 (3.24), His163 (3.55), Ser144 (3.13), and Phe140(3.62)	Met165 (5.09), Cys145 (4.53), and His163 (4.68)	—
1-Methyloctyl Acetate 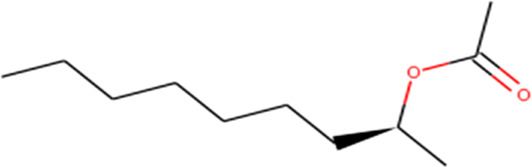	Cys145 (2.34), His163 (2.61, Phe140(3.35), and His163 (2.14)	—	Glu166 (4.78)
Eugenol Acetate 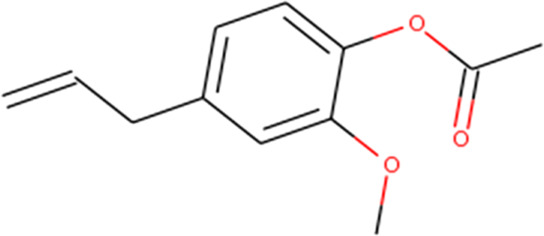	Gln110 (2.18), Asn151(2.25), and Ser158(3.55)	—	—

Our top-ranked compound of eugeniin (−9.8 kcal/mol), syzyginin B (−10.4 kcal/mol), eugenol (−7.3 kcal/mol), and casuarictin (−12.2 kcal/mol) has shown higher binding affinity than the commercially available drugs such as remdesivir (−6 kcal/mol), liponavir (−4 kcal/mol), tamiflu (−5.72 kcal/mol), plaquenil (−5.59 kcal/mol), and mycophenolic (−6.02 kcal/mol) (Arunkumar et al., 2021; Khater and Nassar, 2021). Eugenol is extracted from cloves, is abundantly present in the clove oil, is used for antispasmodic treatment, and acts as a carminative to treat gastrointestinal disorders. They are explored in bioactivities of antioxidant, anti-inflammatory, antiviral, insect-repellent, antimicrobial, and antiparasitic properties with various other related infections (Xu et al., 2016). The additional properties of cloves are strong, pungent, spicy odor and pungent combined aromatic taste, and cloves play a role in industrial application in perfumes, soaps, histological cleaning agents, and anesthetic fishes (Taylor and Roberts 1999). Eugenol exploited against spike glycoprotein for the treatment of SARS-CoV-2 has been reported computationally, with the attempted clinical phase in the official Siddha formulation of Kabasura Kudineer (Kiran et al., 2022). Moreover, the crisis on physical health due to continuous steroid supplementation within individuals infected with the SARS-CoV-2 virus stresses the need for alternative medicine.

### Prediction of Probable Off-Target Activity

By tracing the probable drug reaction within a host and its assumed interaction, it might undergo intruding the host metabolism, which is provided in hit compound target prediction; this paves the way for the preparedness of any drug that can elicit any adverse reaction for patients. Additionally, it provides the researcher with a putative thought to remodel or restrict the further analysis of screened drug-like molecules (Drwal et al., 2014; Daina et al., 2019). For example, eugenol was predicted to be a class 4 toxic substance. The predicted LD50 is 1.930 mg/kg; it does not elicit carcinogenicity, hepatotoxicity, immunotoxicity, mutagenicity, and cytotoxicity and does not interfere with the signaling and stress response pathways. On the other hand, syzyginin B, casuarictin, and eugeniin are classified as class 5 toxic substances and possess an LD50 value of 2.260 mg/kg. This ternion exhibits mild reactivity and less than the recommended level of reactivity toward phosphoprotein (tumor repressor) p53. Still, the concerning part is that it may result in immunotoxicity under unregulated administration. At the same time, the last couple may interfere with the mitochondrial membrane protein and aryl hydrocarbon receptor and may initiate reactions adding up alarm for carcinogenicity, albeit with negligible probability. These results were obtained by combining two toxicity prediction web tools, Swiss Target prediction tools (Gupta et al., 2013). Casuarictin, syzyginin B, and eugeniin are identified to have common off-target interactions whose extent of integration either overtaking the active antagonist property or least significant can be identified only upon wet-lab studies (Mohamed et al., 2021). The common targets of these three compounds include beta secretase, squalene mono-oxygenase, thrombin and coagulation factor, troponin, and cardiac muscles with varying proportions (Figure 4).

### Molecular Dynamics Simulation

**FIGURE 5:**
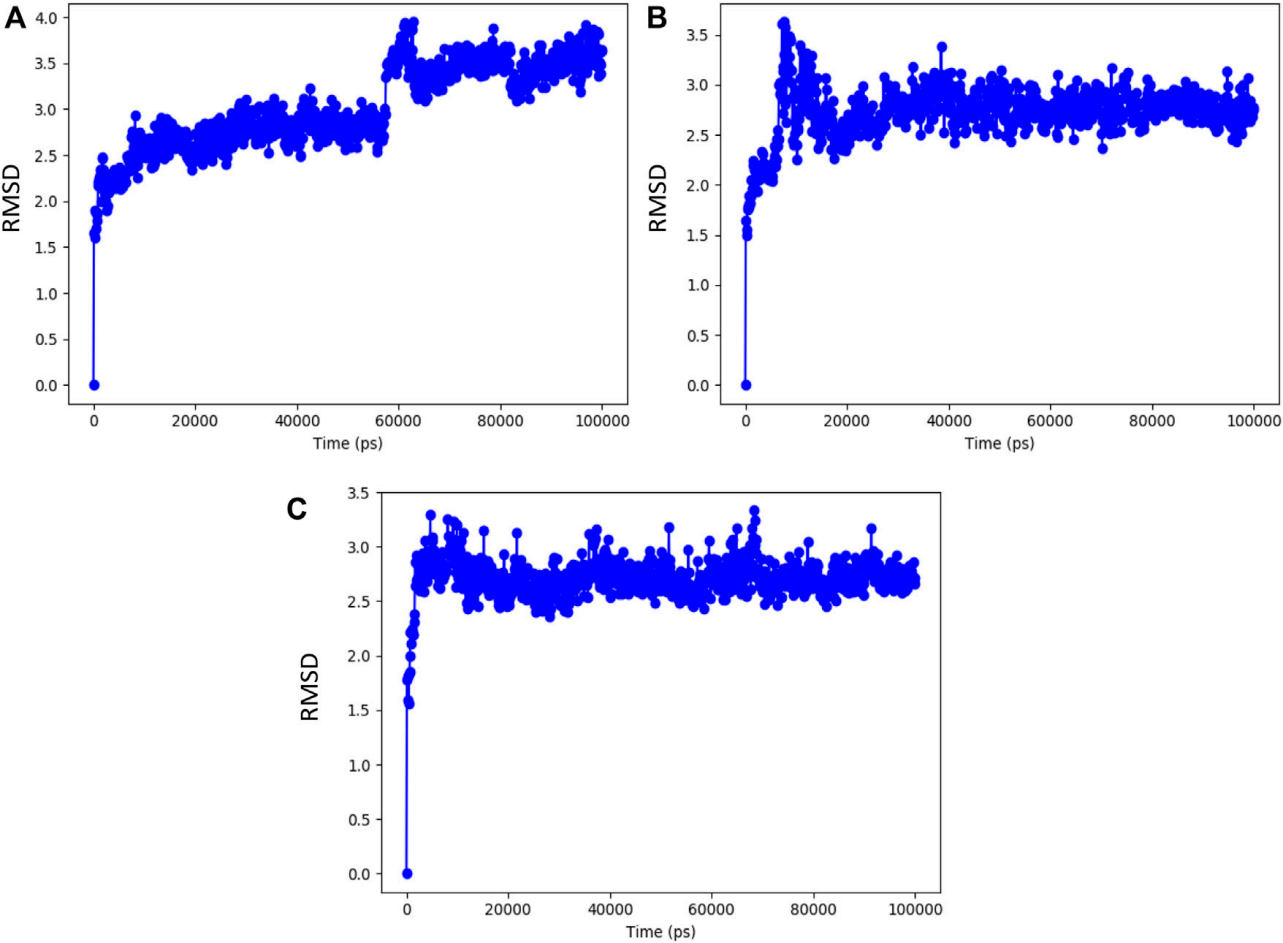
RMSD value of the complex molecule of the main protease with **(A)** syzyginin B, **(B)** eugeniin, and **(C)** eugenol.

The resulting top-ranked docking complex molecule was considered for performing the molecular dynamics simulation for further validation. In these simulation studies, the protein interaction with the ligand molecules was studied throughout the total simulation time of 100 ns with 1,000 projection points (Frames). The macromolecules and ligand causing interactions throughout the simulation time are called contacts, classified based on hydrogen bonds, hydrophobic interactions, ionic bonds, and water bridges. The molecular dynamics simulation output was investigated with a root-mean-square deviation (RMSD) value around 3 Å distance, representing the stability of the complex molecule. Syzyginin B starts the stability at around 60 ns in the first phase with the deviation of around 1 Å distance and remains stable around 2.5 Å. After that, it deviates from the cavity site of 1 Å distance, which holds the stability in 3.5 Å up to a total simulation time of 100 ns. On the other hand, eugeniin deviates in the initial phase up to 3.5 Å and stabilizes with minimal deviation, which remains stable from 20 to 100 ns around 2.8 Å RMSD. Finally, eugenol started the initial phase around 2.9 Å and remains stable across 100 ns of the total simulation time within 2.8 Å. Moreover, all the three complex molecules show better results from the molecular dynamics simulation studies, depicted by the graph in Figure 5.

The protein–ligand contact of clove phytochemical eugeniin showed an interaction of discontinuous contacts in the active site amino acid of LYS5, GLY170, SER139, and PHE140 and continuous contacts with GLU166, LYS137, and GLN27 across the total simulation time. On the other hand, the remaining two complex molecules of syzyginin B and eugenol showed the major discontinuous contacts (Supplementary Figures S20–22).

### Pharmacokinetics

Computational ADMET screening can reduce the cost of high capital-consuming wet-lab trials that may end up in failure on many occasions (Hage-Melim et al., 2020; Tongqiang Zhang, et al., 2020). In the current study, not all the selected molecules were following the optimal limit of ADMET properties. With the octanal/water partition coefficient, aqueous solubility, and brain/blood partition coefficient, all compounds align within an acceptable range of −2.0–6.5, −6.5–0.5, and −3.0-2.1, respectively (Gleeson et al., 2009). A major deviation was exhibited by casuarictin (−13.959) and euginiin (−13.959) for skin permeability, where the optimum range is between −8.0 and −1.0. Eugenol exhibited a minimum metabolic reaction limited to 3, followed by protopine with five reactions. At the same time, the next two molecules deviate from an acceptable band of 1–8, where both euginiin and casuarictin exhibit 15 reaction exceptions.

All molecules are efficient enough to bind with human serum albumin within the accepted range of −1.5 to 1.5. Eugenol and protopine are highly capable of oral absorption and hold a good van der Waals surface area of polar nitrogen and oxygen atoms and carbonyl carbon atoms. Euginiin and casuarictin initially exhibited high binding energy (mostly negative). However, they face the most number of violations, albeit their ability to infuse toxicity within an individual is low, which suggests that its efficacy can be taken for the next level of analysis as hit compounds (Liao and Nicklaus, 2009; Zhong et al., 2013) (Table 2).

**TABLE 2:** ADME/T properties of various compounds that exhibited a high binding efficiency.

Title (and Range)	Syzyginin (ZINC230067171)	Syzyginin B	Eugeniin	Casuarictin	Protopine	Eugenol
#amine (0–1)	0	0	0	0	1	0
#amidine (0)	0	0	0	0	0	0
#acid (0–1)	0	0	0	0	0	0
#amide (0–1)	0	0	0	0	0	0
CNS (-2 inactive and +2 active)	-2	-2	-2	-2	2	0
mol MW (130.0–725.0)	756.54	756.54	938.672	936.657	353.374	164.204
Dipole (1.0–12.5)	3.342	7.316	4.159	10.409	2.299	1.859
SASA (300.0–1,000.0)	915.82	924.397	1,186.696	1,133.803	508.221	401.412
FOSA (0.0–750.0)	105.614	97.55	66.353	76.301	310.695	204.373
FISA (7.0–330.0)	612.263	621.364	835.169	810.688	26.505	51.309
PISA (0.0–450.0)	197.944	205.484	285.174	246.814	171.021	145.731
WPSA (0.0–175.0)	0	0	0	0	0	0
Volume (500.0–2000.0)	1786.437	1800.373	2,304.514	2,217.198	973.528	641.616
donorHB (0.0–6.0)	12	12	15	14	0	1
accptHB (2.0–20.0)	19.6	19.6	22.95	21.95	7	1.5
dip^2/V (0.0–0.13)	0.006252	0.029726	0.007507	0.048864	0.005431	0.005386
ACxDN^.5/SA (0.0–0.05)	0.074137	0.073449	0.074901	0.072437	0	0.003737
Glob (0.75–0.95)	0.777,464	0.774,251	0.711,015	0.725,266	0.934,713	0.89624
QPpolrz (13.0–70.0)	58.24	58.87	75.312	74.02	34.44	18.347
QPlogPC16 (4.0–18.0)	23.298	23.568	31.713	29.985	9.054	5.793
QPlogPo/w (-2.0–6.5)	-2.911	-2.905	-3.469	-3.237	1.727	2.666
QPlogS (-6.5–0.5)	-3.622	-3.716	-4.733	-5.061	-0.963	-2.35
QPPCaco (<25 poor, >500 great)	0.015	0.013	0	0	1,385.006	3,231.023
QPlogBB (-3.0–1.2)	-7.033	-7.191	-11.716	-10.736	0.76	-0.103
QPPMDCK (<25 poor, >500 great)	0.003	0.003	0	0	778.255	1757.464
QPlogKp (-8.0 to −1.0)	-10.762	-10.903	-13.891	-13.959	-3.468	-1.568
IP(eV) (7.9–10.5)	8.542	8.383	9.017	8.897	8.923	8.729
EA (eV) (-0.9–1.7)	0.881	0.658	0.634	0.737	0.406	-0.232
QPlogKhsa (-1.5–1.5)	-1.035	-1.025	-1.246	-0.984	-0.428	-0.113
Human Oral Absorption	1	1	1	1	3	3
Percent Human Oral Absorption (>80% is high, <25% is poor)	0	0	0	0	93.285	100
RuleOfFive (maximum is 4)	3	3	3	3	0	0
RuleOfThree (maximum is 3)	2	2	2	2	0	0

The ability of candidates to get accommodated within the traditional laws of drug-likeliness and physiochemical property limits are major initial screening processes, aiming to screen out unfit candidates (Mohamed et al., 2021). For example, Lipinski’s rule of five (RO5) is a major factor for drug-likeness that aids in identifying the potential compounds from a pool of drug-like molecules which must have strong gastrointestinal absorption, high oral bioavailability, and descent membrane permeability, with their log *p* ≤ 5; MW ≤ 500 Da, HBDs ≤5, and HBAs ≤10 (Gleeson et al., 2009). Natural compounds that have already been identified for treatment purposes are reported to violate RO5. On the other hand, due to the significant efficacy of natural compounds, those from marine-based and terrestrial resource-based compounds are accepted, although they have been identified as violating RO5 (Zhong et al., 2013; Mohanraj et al., 2018). The new framework proposed by the FDA over relaxed and diluted norms for FDA approval of drugs supports the idea to migrate the sample for further intensive trials so as to find the therapeutic scope beyond RO5.

Violation of log P is still not a concern for major cancer drugs (DeGoey et al., 2018). Casuarictin violates Veber’s rule for polar surface area (TPSA), resulting in a range ≤ 140 Å by holding a value of 444.18. The existing drugs that are prescribed for being effective against COVID-19 virus-like lopinavir, ritonavir, and remdesivir also violate Lipinski’s RO5, where lopinavir disobeys with MW > 500, many rotatable bonds being >10, ritonavir and remdesivir at features like MW > 500, many rotatable bonds > 10, and TPSA > 140 Å (Kar et al., 2021). Despite few notable fluctuations expressed by these selected compounds, there are updated relaxed norms by FDA over exploiting potential drugs that still disobey drug-likeliness properties and the existing record of supplementing drugs of a high molecular weight, disobeying RO5 (DeGoey et al., 2018).

## Conclusion

Using biological sources to find alternative and successful drug candidates could be a long-term strategy for improving the COVID-19 drug discovery process. Cloves are a rich source of bioactive chemicals such as eugenol, which have been shown to have antiviral and immunostimulatory activities. In the current computational approach, clove phytochemicals of a total of 53 compounds were investigated for the molecular docking experiment against the M^Pro^ of SARS-CoV-2. Interestingly, among them, eugeniin, syzygininB, eugenol, and casuarictin have shown possible antagonist properties against the M^Pro^ with significant binding energies. Furthermore, the top-ranked phytochemicals were validated with the molecular dynamics simulation and revealed three compounds, namely, syzyginin B, eugeniin, and eugenol, as strongly interacting compounds that got stabilized with the least deviation from the site of interaction over the observed total simulation time. Moreover, the phytochemicals were assessed for their pharmacokinetic properties, shown to be druggable with no significant violation of any ADMET profiling parameters. As a result, these cloves’ phytochemicals may be viable candidates against SARS-CoV-2. Eugenol is one of the formulations of official siddha as Kabasura Kudineer for the treatment of COVID-19. However, extensive research is required to determine its efficacy as an antiviral drug, particularly *in vitro* trials against SARS-CoV-2. Finally, the innovative findings of this study could have a significant impact on the advancement of COVID-19 antiviral drug interventions in the near future (Trott and Olson, 2010; Lin et al., 2020; Khanna et al., 2021).

## Data Availability

The original contributions presented in the study are included in the article/Supplementary Material. Further inquiries can be directed to the corresponding authors.
