# Supplementary material for: Evaluation of Clove Phytochemicals as Potential Antiviral Drug Candidates Targeting SARS-CoV-2 Main Protease: Computational Docking, Molecular Dynamics Simulation, and Pharmacokinetic Profiling
Source: Front Mol Biosci. 2022 Jun 28;9:918101. doi: 10.3389/fmolb.2022.918101 (PMC9273859; doi:10.3389/fmolb.2022.918101)
Supplement: Supplementary file 1 [file DataSheet1.PDF]

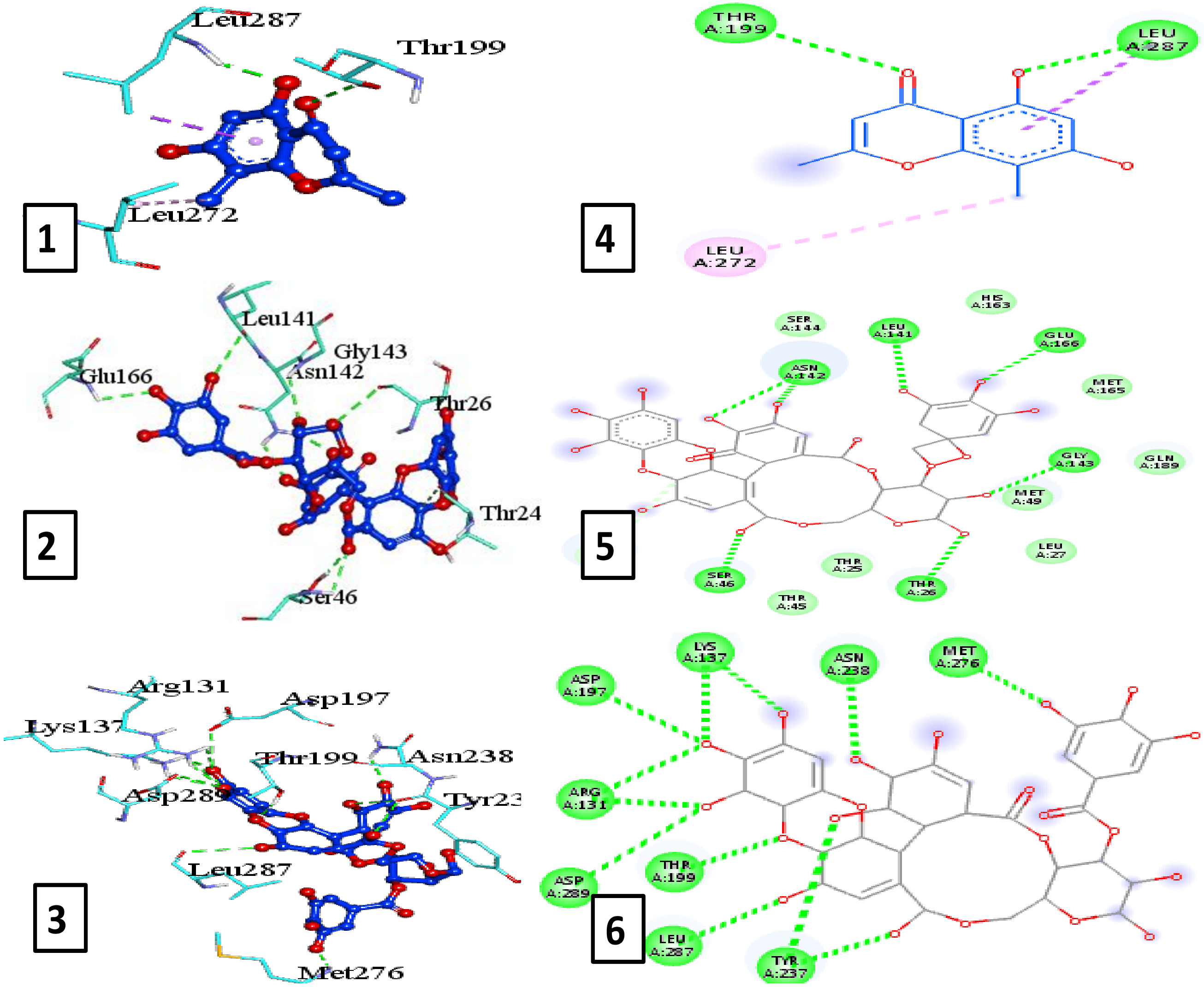

Supplementary Figure S 1: The three-dimensional interaction of the main protease with the ligand molecules of 1.Isoeugenitol, 2.Syzyginin B, 3.Ellagic acid di-hexoside, and 4, 5, 6 are their corresponding 2-D interaction

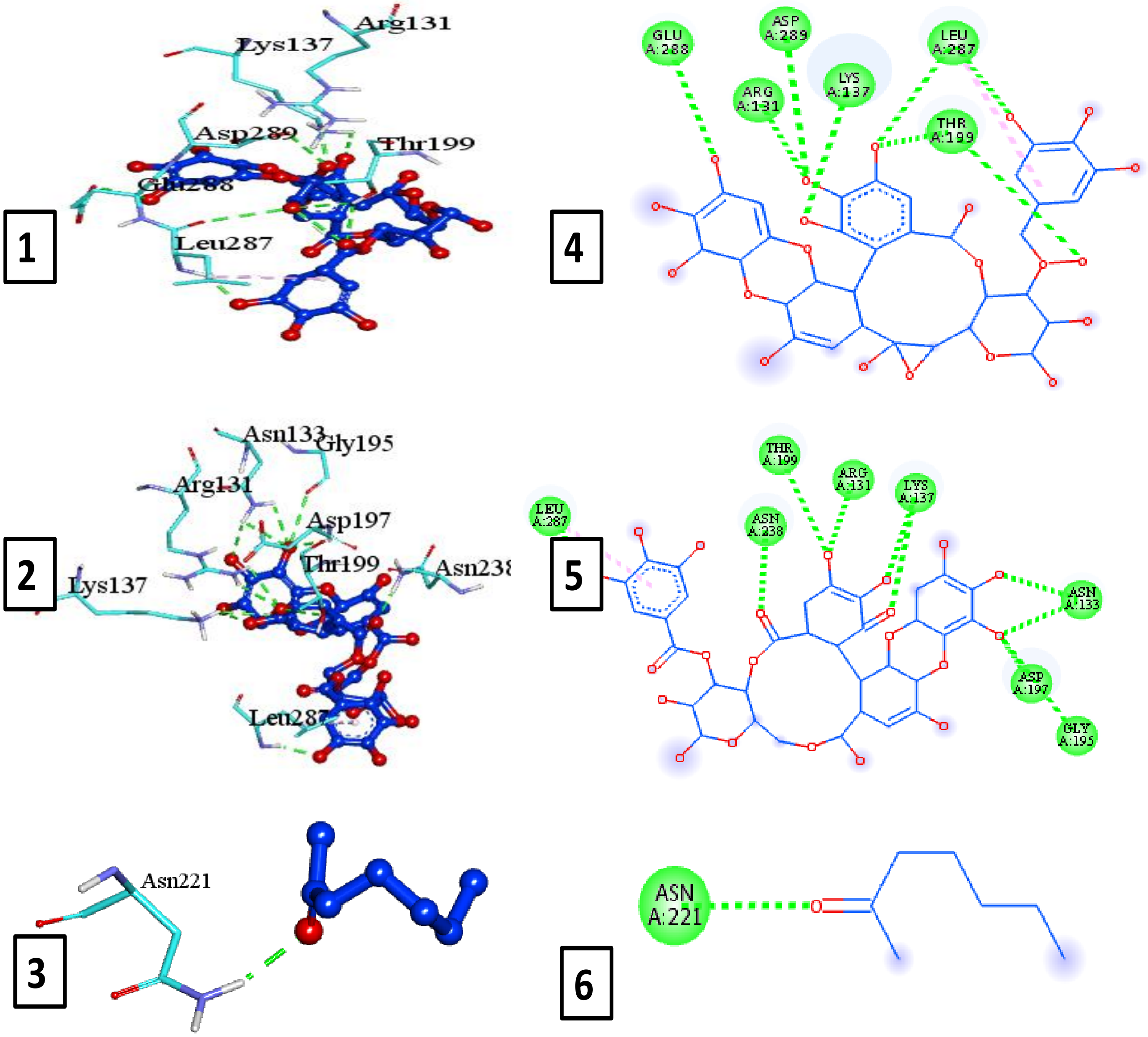

Supplementary Figure S 2: The three-dimensional interaction of the main protease with the ligand molecules of 1. Nobotanin D, 2. Punicalgin, 3. 2 Hrptanone and 4, 5, 6 are their corresponding 2-D interaction

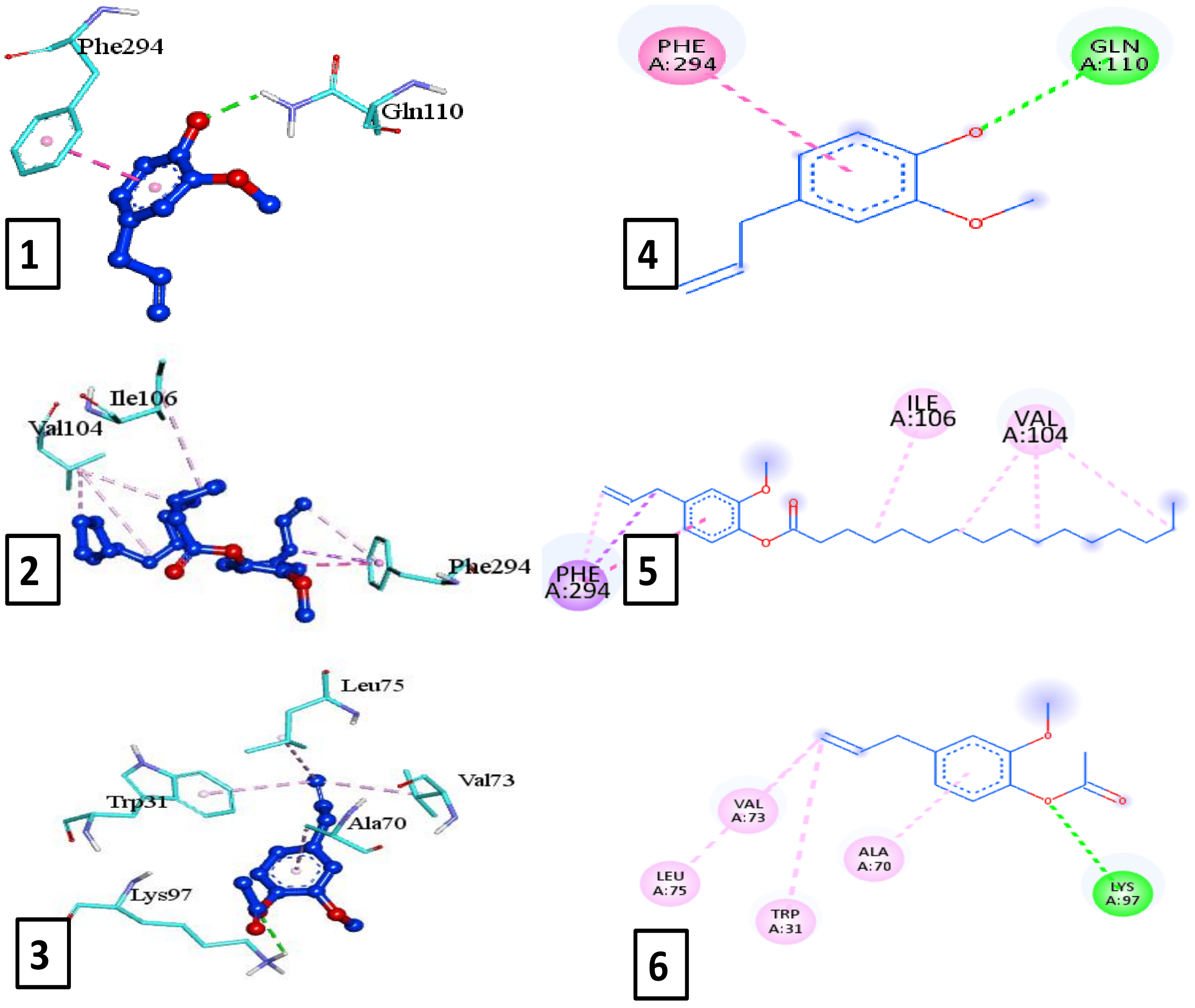

Supplementary Figure S 3: The three-dimensional interaction of the main protease with the ligand molecules of 1. Eugenol, 2. Eugenol Acetate, 3. 2-methoxy-4-propyl phenol and 4, 5, 6 are their corresponding 2-D interaction

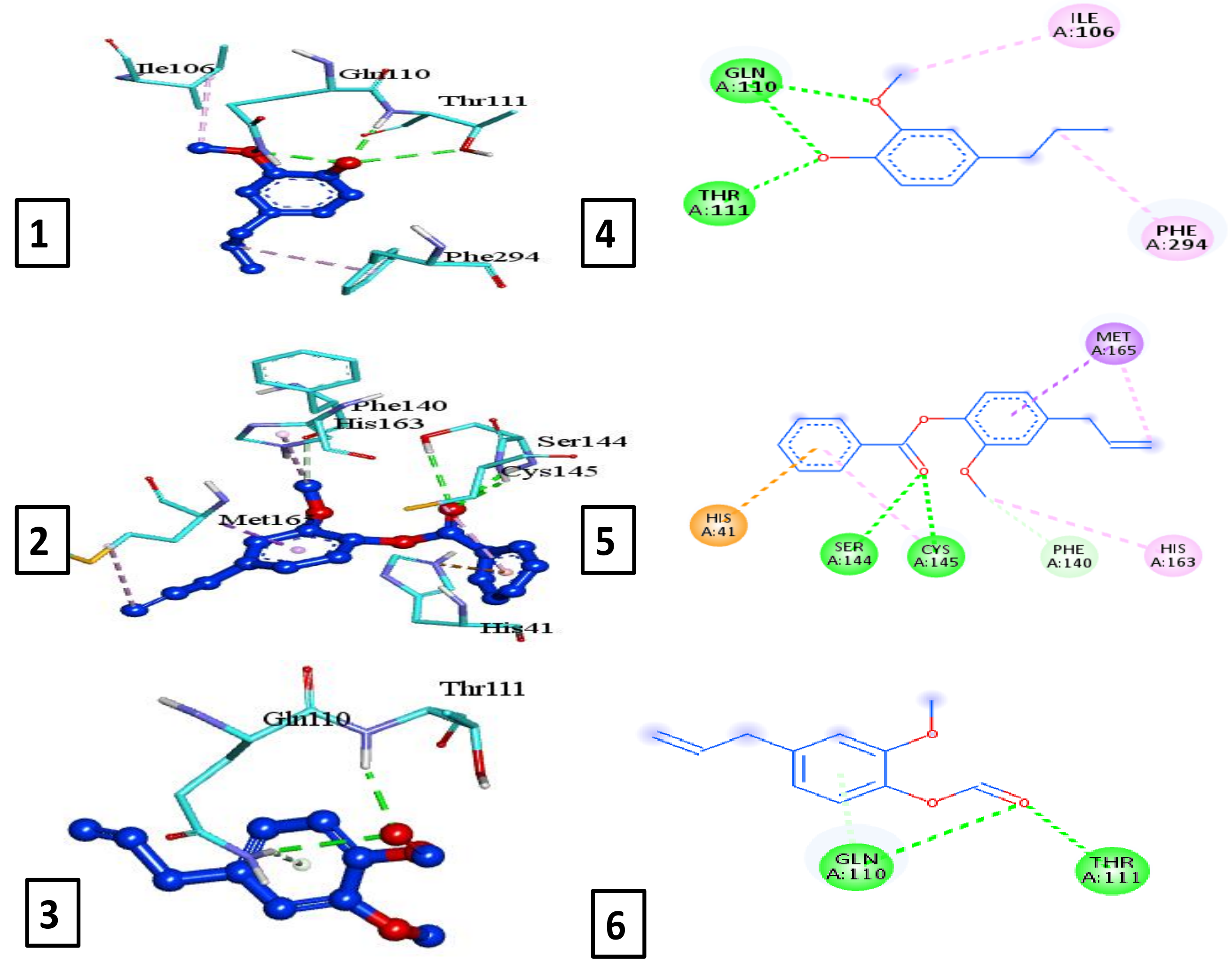

Supplementary Figure S 4: The three-dimensional interaction of the main protease with the ligand molecules of 1. Eugenyl Benzoate, 2. Vanilloloside, 3. EugenylFormate and 4, 5, 6 are their corresponding 2-D interaction

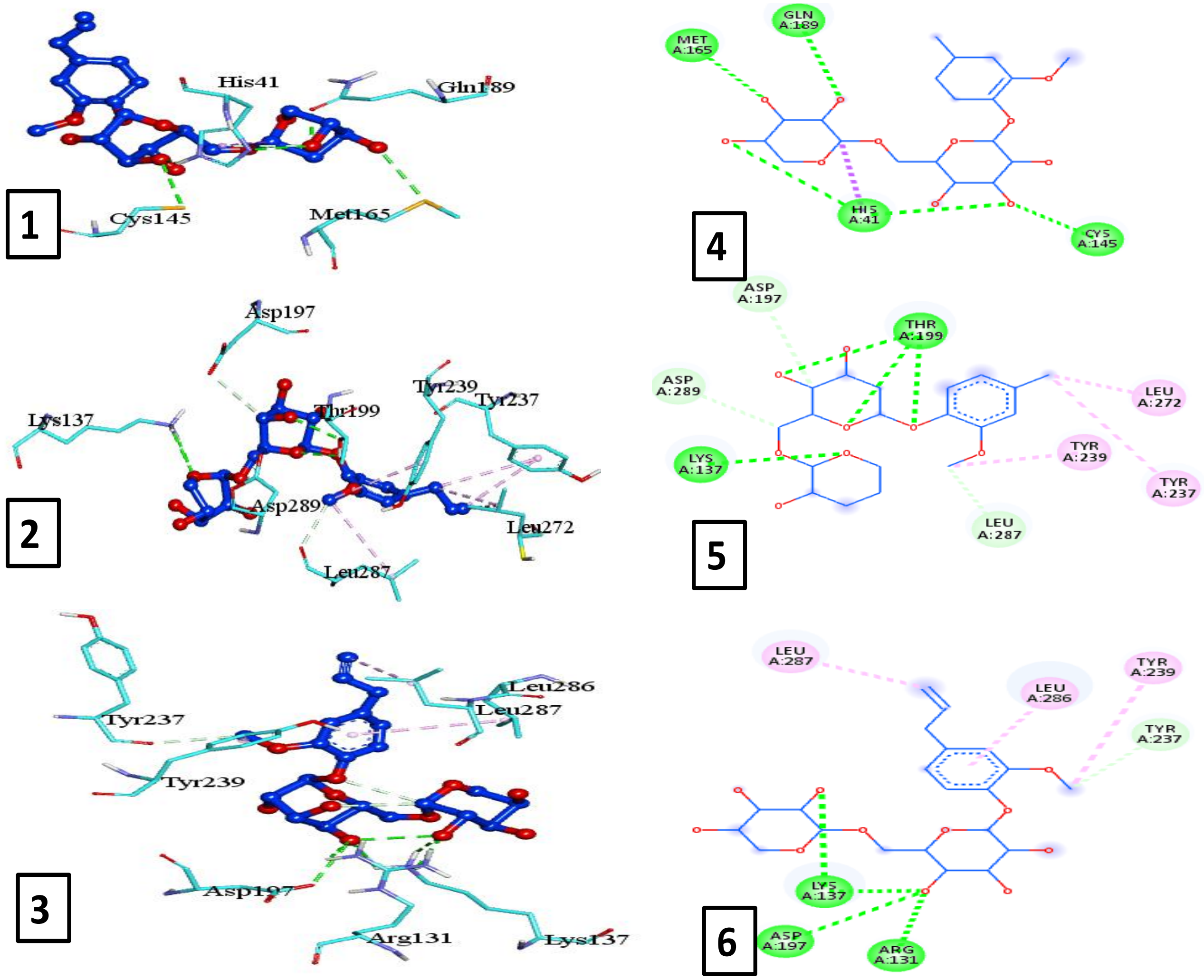

Supplementary Figure S 5: The three-dimensional interaction of the main protease with the ligand molecules of 1. DMPX, 2. BS-DMPX, 3. MS-DMPX and 4, 5, 6 are their corresponding 2-D interaction

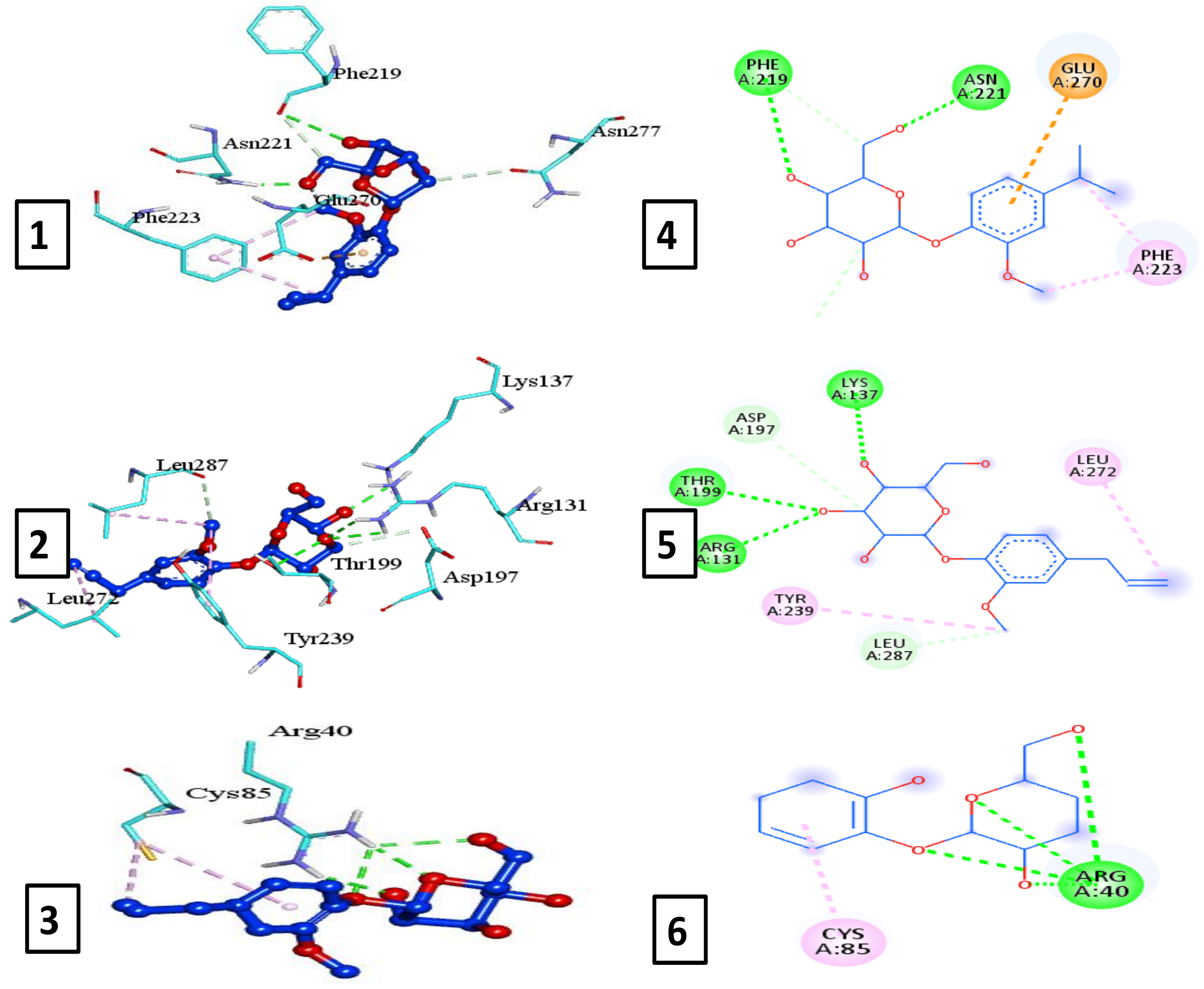

Supplementary Figure S 6: The three-dimensional interaction of the main protease with the ligand molecules of 1. Eugenyl-GX, 2. Syringin, 3. Eugenol Glucoside and 4, 5, 6 are their corresponding 2-D interaction

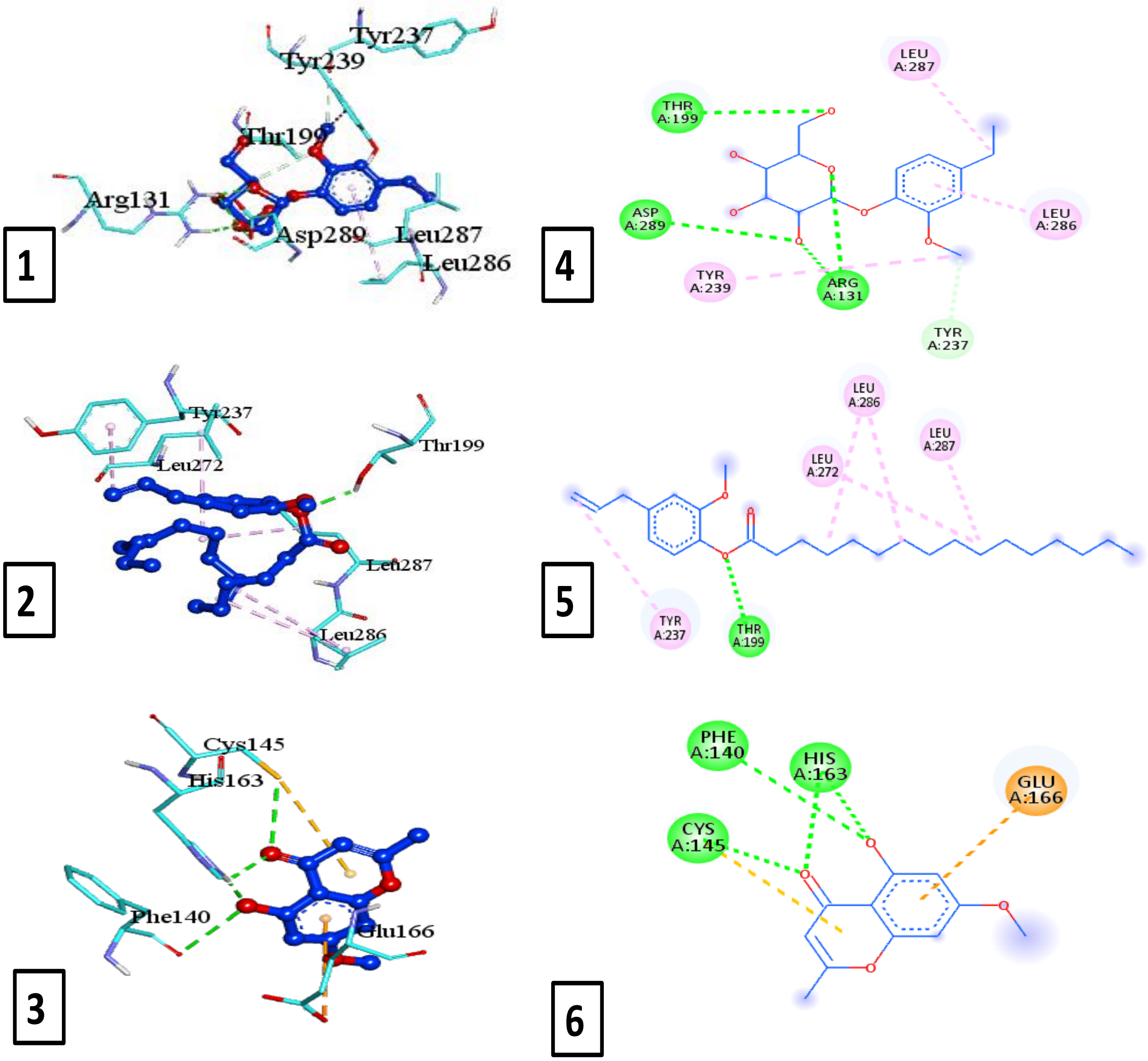

Supplementary Figure S 7: The three-dimensional interaction of the main protease with the ligand molecules of 1. Citrusin D, 2. Pentagalloylglucose, 3. Eugeniin and 4, 5, 6 are their corresponding 2-D interaction

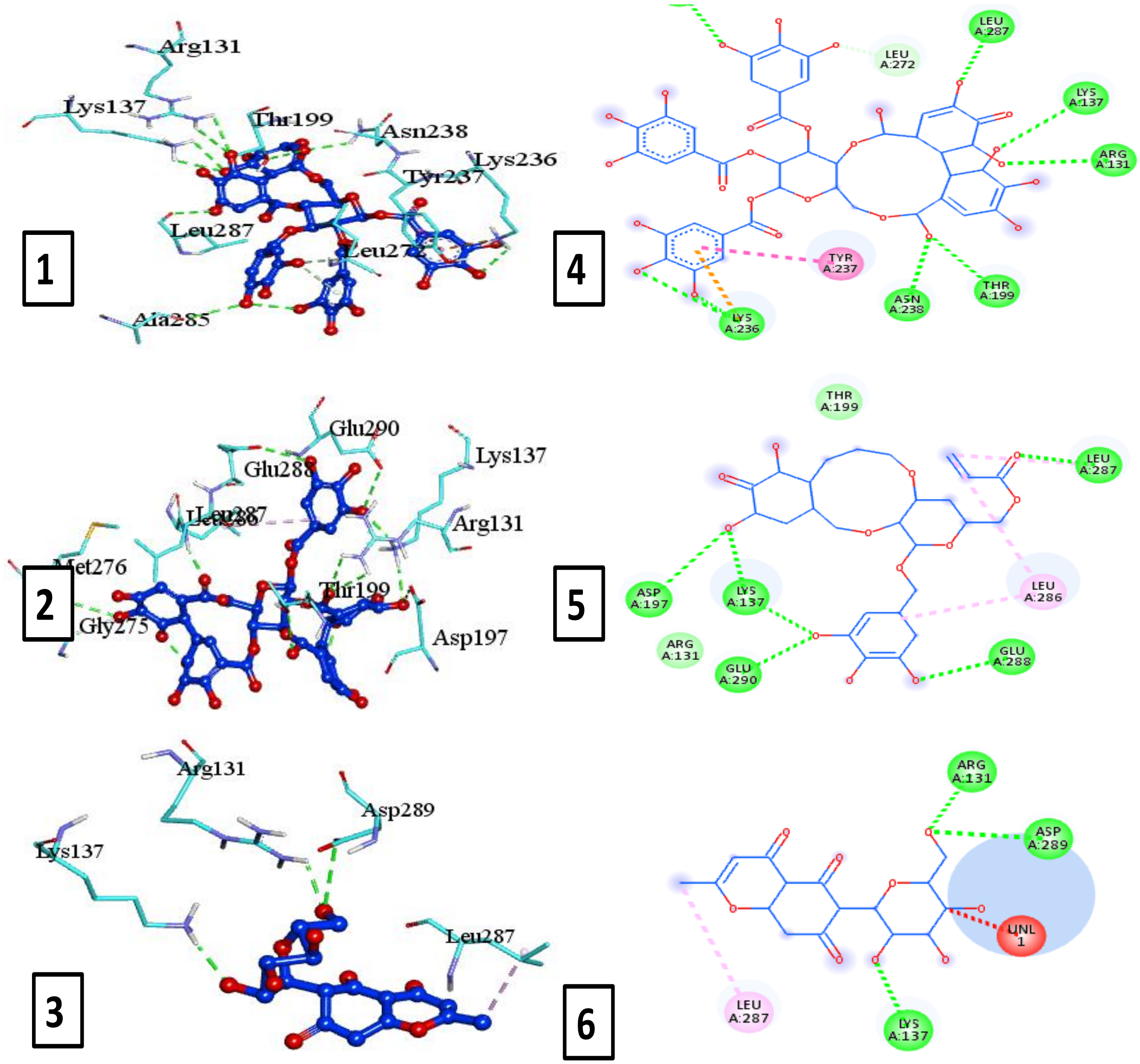

Supplementary Figure S 8: The three-dimensional interaction of the main protease with the ligand molecules of 1. Casuarictin, 2. 2 Heptyl Benzoate, 3. Biflorin and 4, 5, 6 are their corresponding 2-D interaction

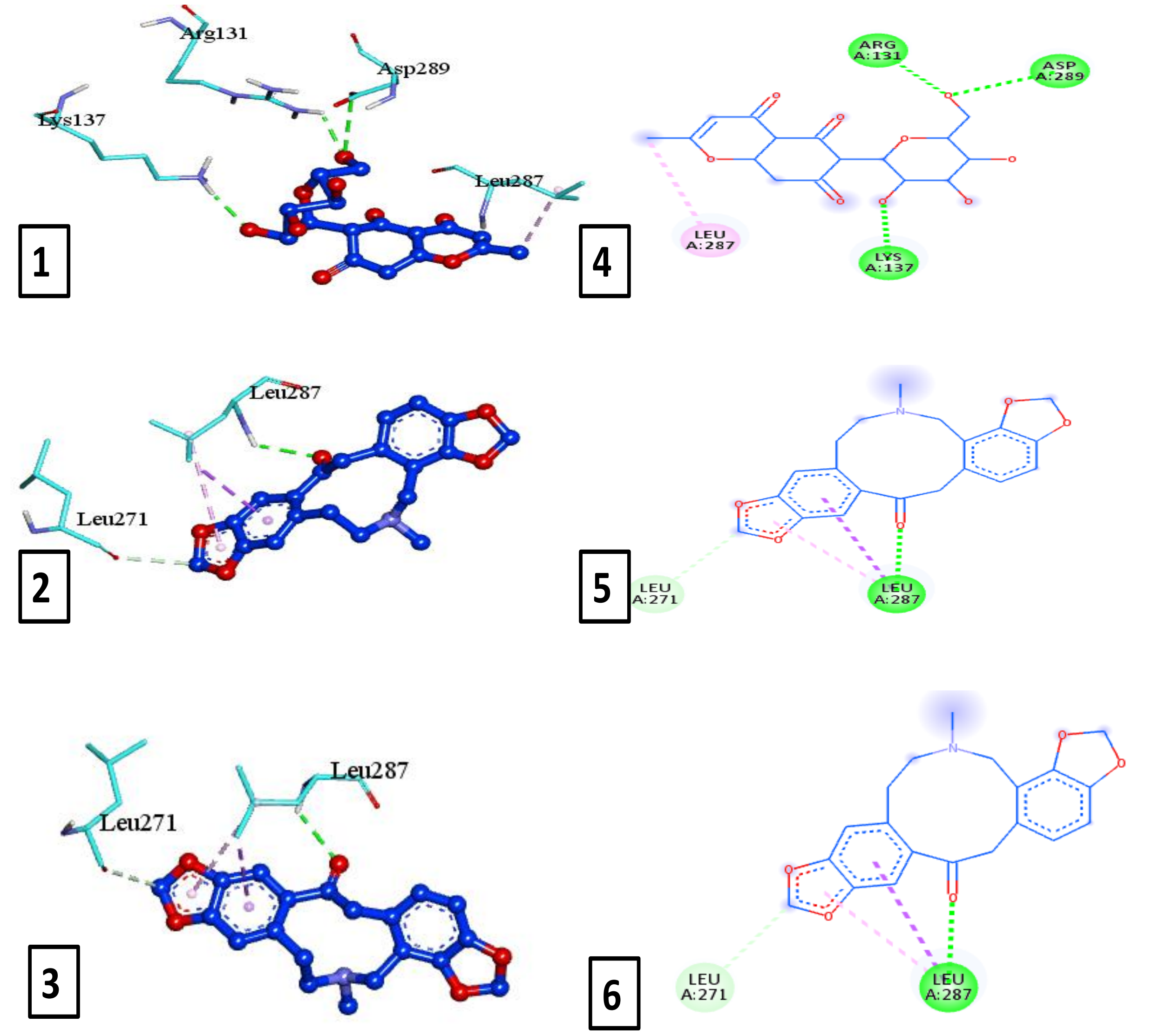

Supplementary Figure S 9: The three-dimensional interaction of the main protease with the ligand molecules of 1. Protopine, 2. Digalloylglucose, 3. 2-[(Galloyloxy)Methyl]-Alpha-D-Ribofuranose 5-(3,4,5-Trihydroxybenzoate) and 4, 5, 6 are their corresponding 2-D interaction

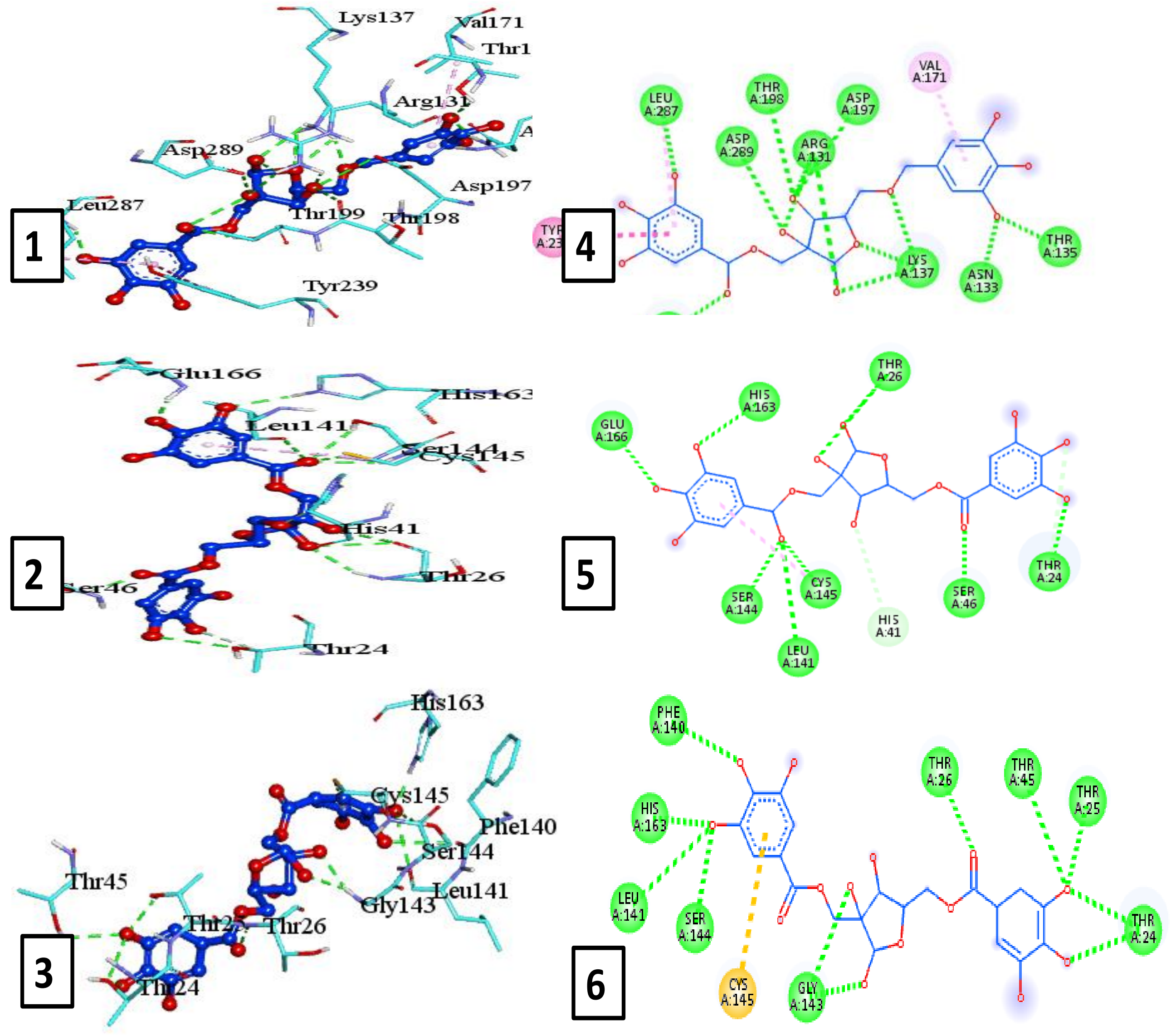

Supplementary Figure S 10: The three-dimensional interaction of the main protease with the ligand molecules of 1. Hamamelitannin, 2. 1,6- Digalloylglucose, 3. 2, 6 –Digalloylglucose and 4, 5, 6 are their corresponding 2-D interaction

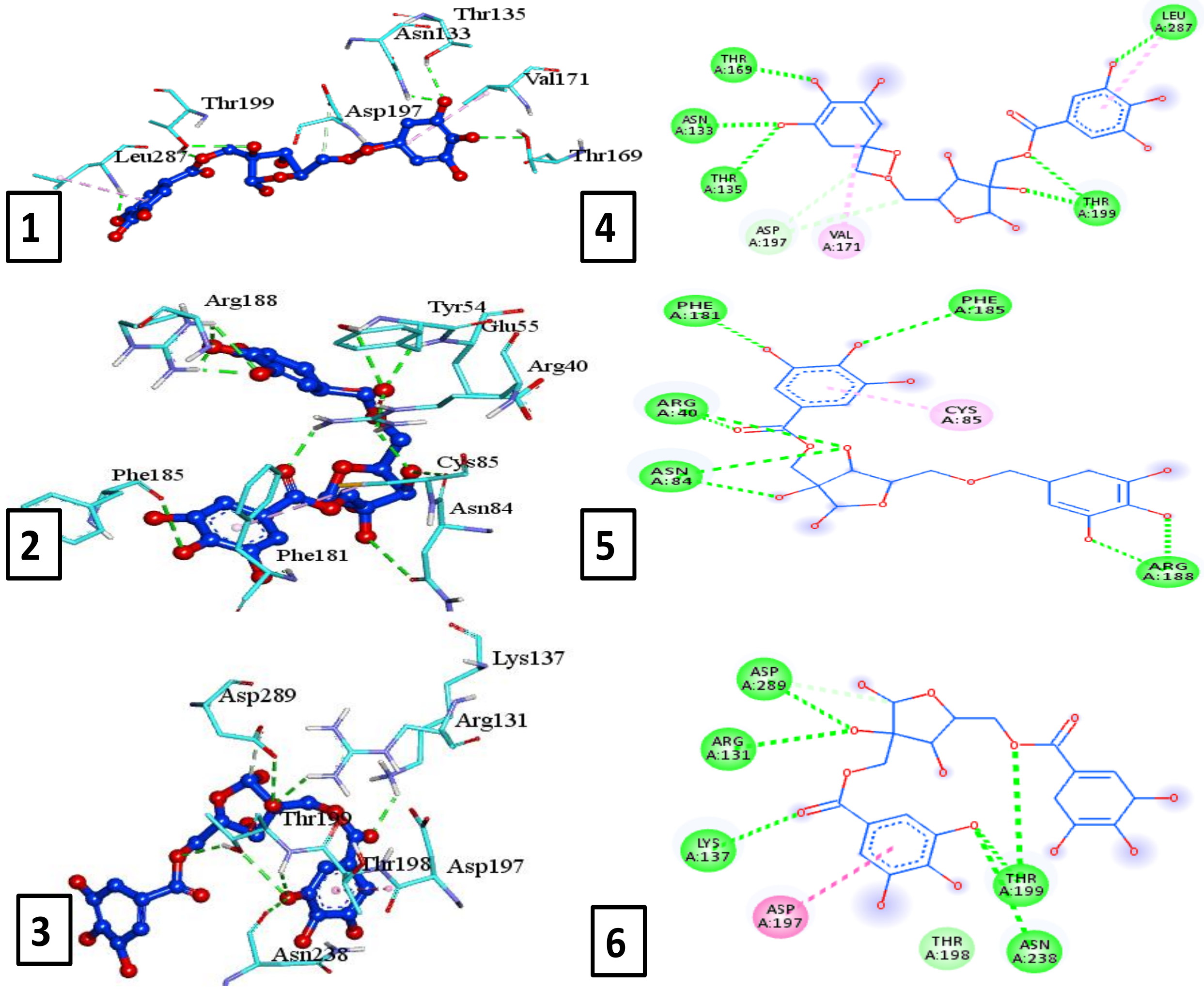

Supplementary Figure S 11: The three-dimensional interaction of the main protease with the ligand molecules of 1. 1,2-Digalloylglucose, 2. 1,6-Bis-O-Galloyl-Beta-D-Glucose; 3. (2S)-nonan-2-ol and 4, 5, 6 are their corresponding 2-D interaction

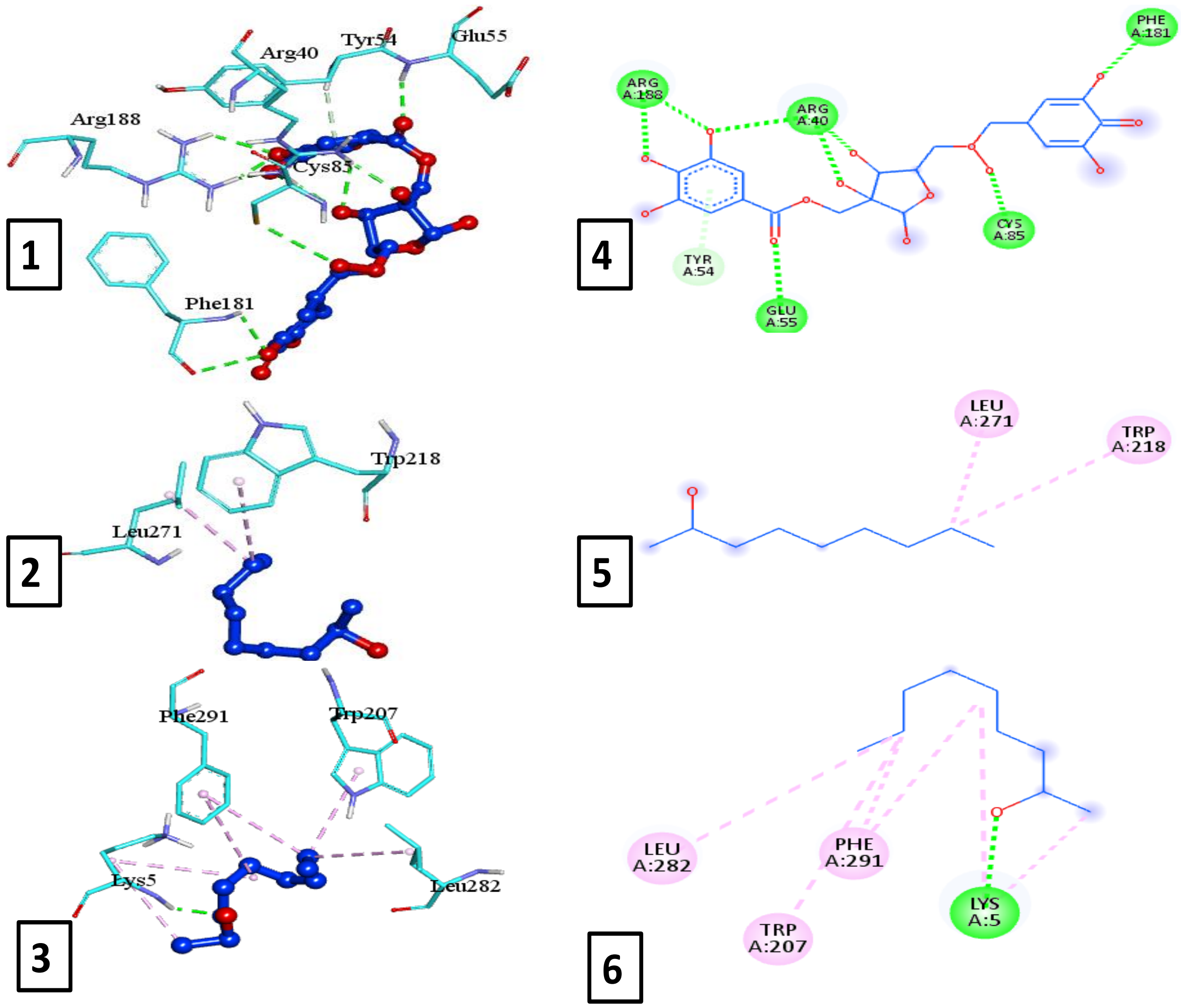

Supplementary Figure S 12: The three-dimensional interaction of the main protease with the ligand molecules of 1. Nonan-2R-Ol, 2. Acetyleugenol, 3. 1-Methyloctyl Acetate and 4, 5, 6 are their corresponding 2-D interaction

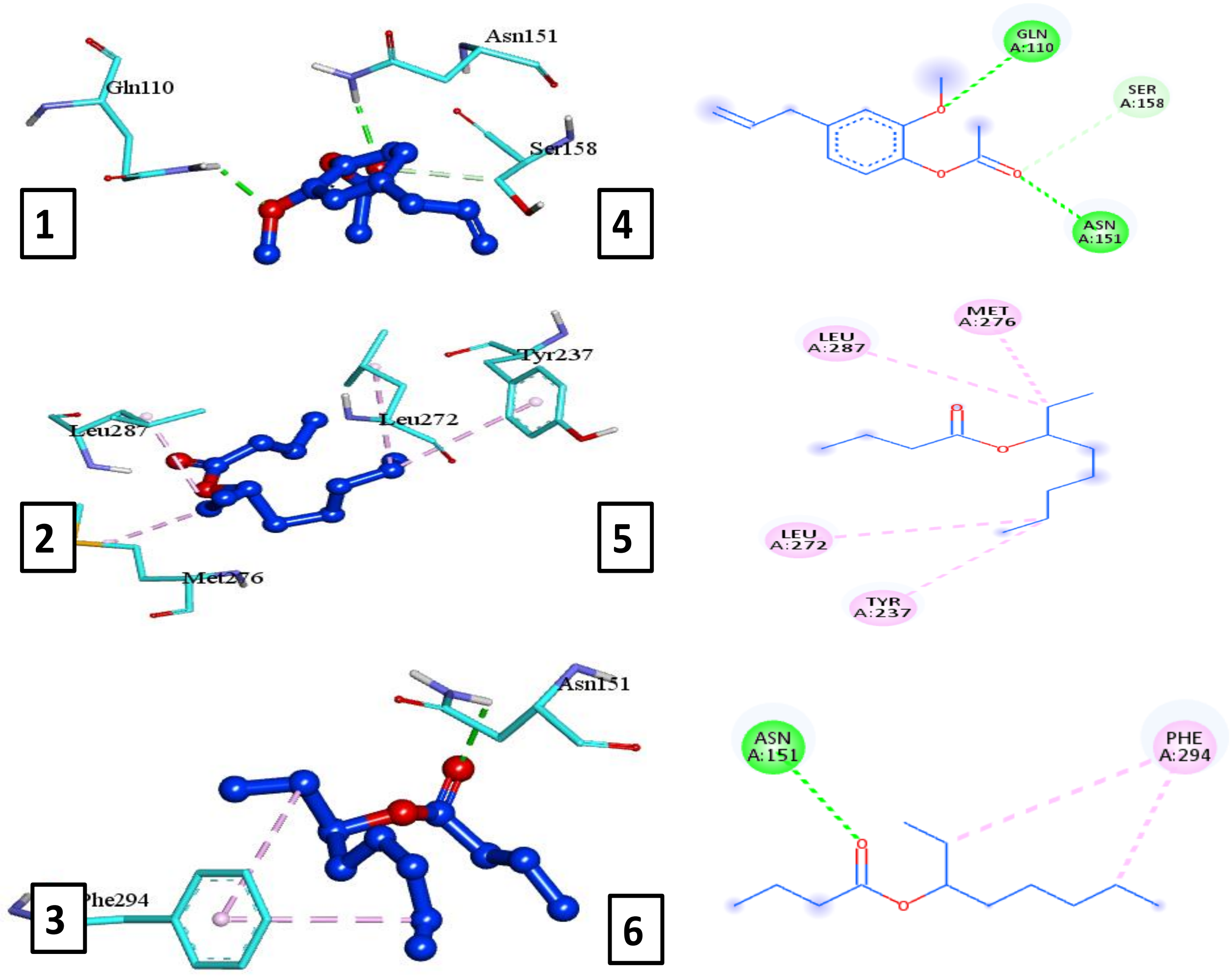

Supplementary Figure S 13: The three-dimensional interaction of the main protease with the ligand molecules of 1. 2-Heptanone, 2. [(3S)-octan-3-yl] butanoate, 3. [(3R)-octan-3-yl] butanoate and 4, 5, 6 are their corresponding 2-D interaction.

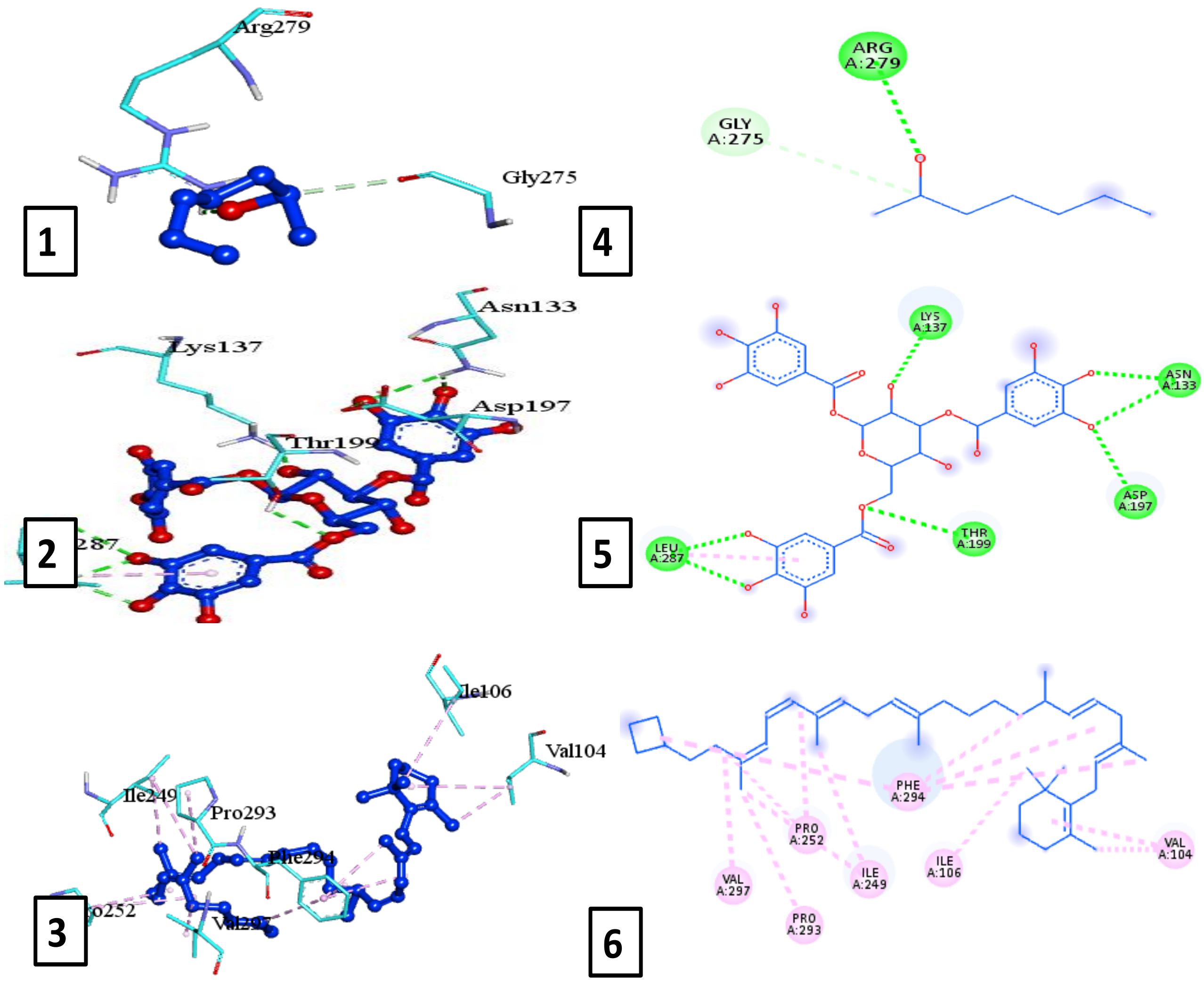

Supplementary Figure S 14: The three-dimensional interaction of the main protease with the ligand molecules of 1. Crategolic Acid, 2. 1,3,6-Tri-O-Galloyl-B-D-Glucopyranose, 3. 6,10,10-Trimethyl-2-Methylenebicyclo(7.2.0)Undec-5-En-3-Ol and 4, 5, 6 are their corresponding 2-D interaction

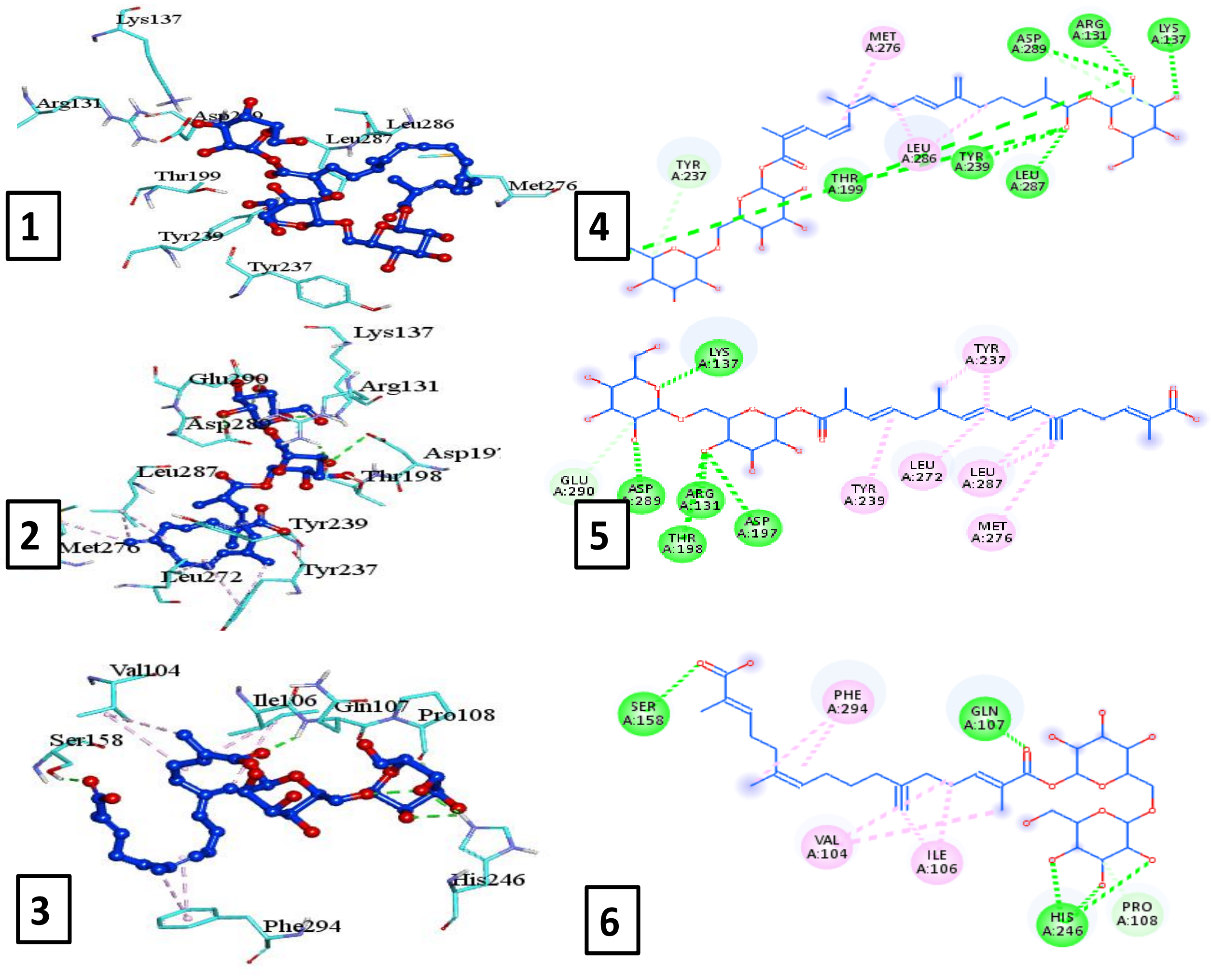

Supplementary Figure S 15: The three-dimensional interaction of the main protease with the ligand molecules of 1. 1-Decanol, 2. Beta Carotene, 3. Friedlein and 4, 5, 6 are their corresponding 2-D interaction

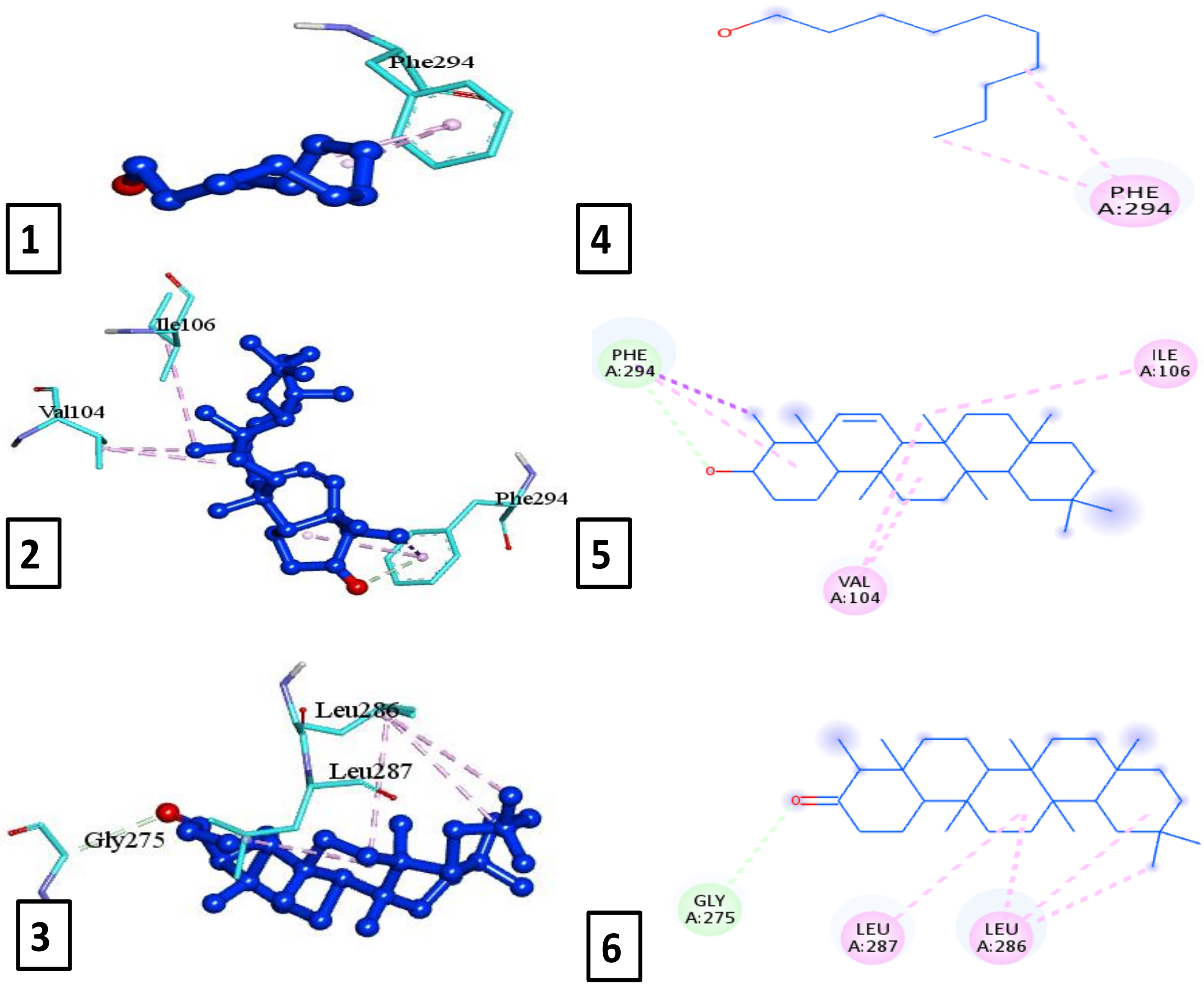

Supplementary Figure S 16: The three-dimensional interaction of the main protease with the ligand molecules of 1. 4-Epi-Friedelin, 2. Galactiol, 3. Hetastarch and 4, 5, 6 are their corresponding 2-D interaction

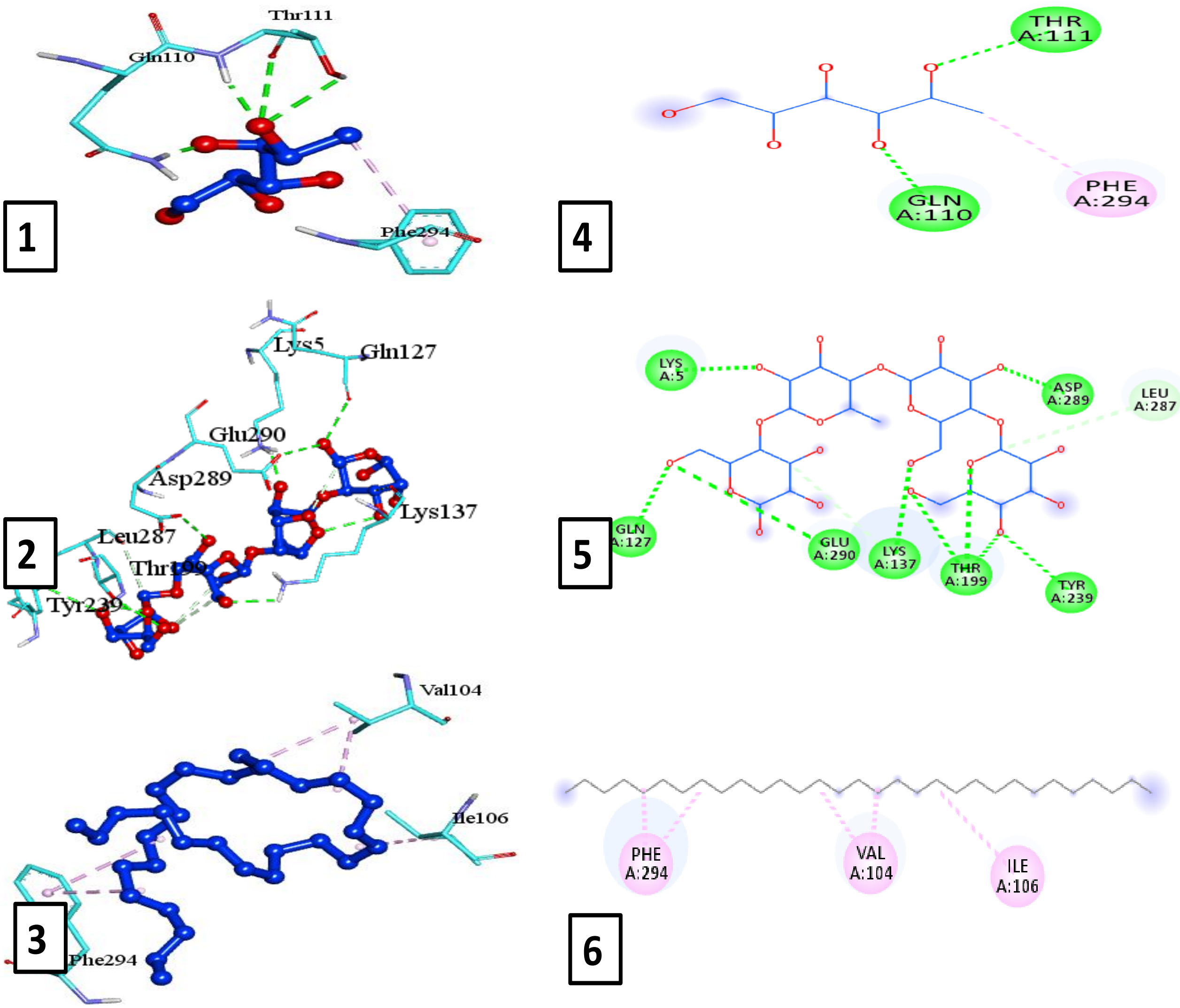

Supplementary Figure S 17: The three-dimensional interaction of the main protease with the ligand molecules of 1. Hentriacontane, 2. Ascorbic Acid, 3. 6-O-Stearoyl-L-Ascorbic Acid, and 4, 5, 6 are their corresponding 2-D interaction

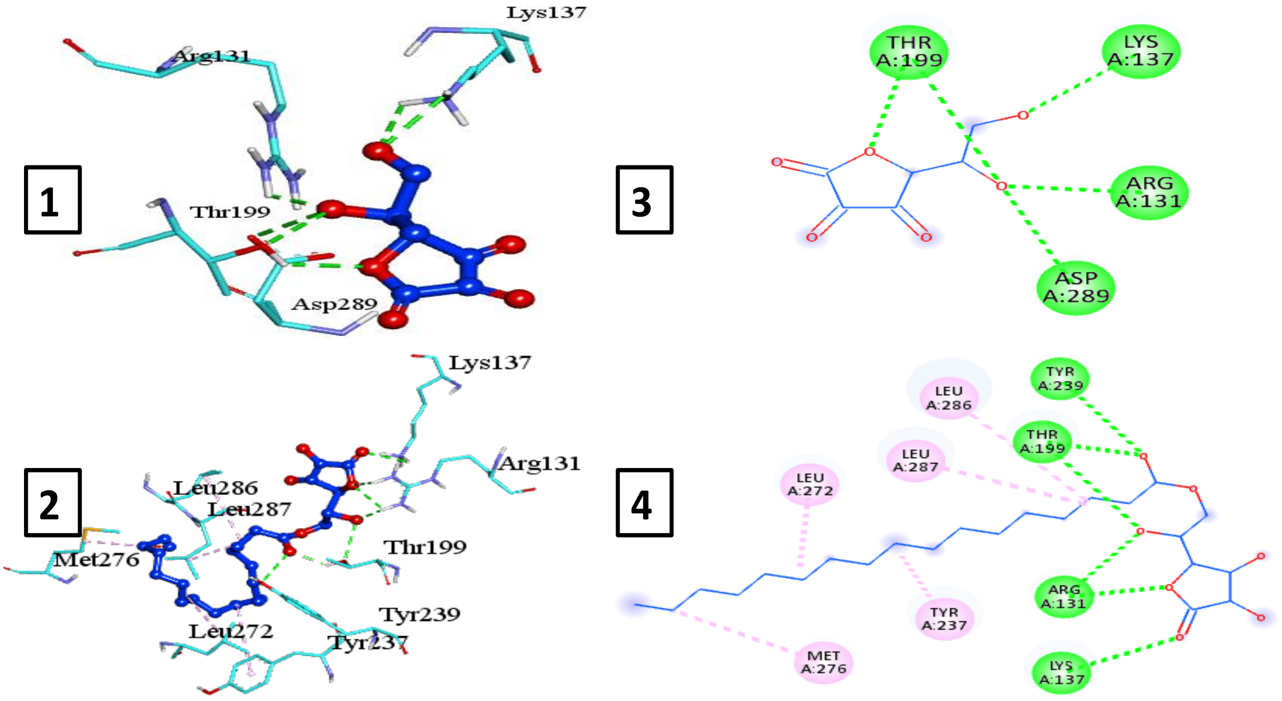

Figure 18: The three-dimensional interaction of the main protease with the ligand molecules of 1. Syringoside, and 2. Oryzarol, and 3, 4 are their corresponding 2-D interaction, and 4, 5, 6 are their corresponding 2-D interaction

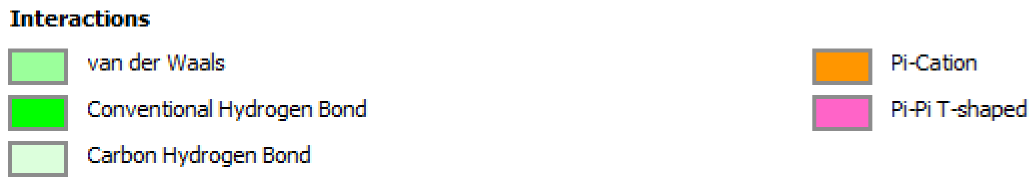

Supplementary Figure S19: overall legends of the 2D interaction graph.

Supplementary Table S1: Interacting residues of all 53 compounds within binding site at Covid-19 main protease and amino acids forming hydrogen bonds:

S. No	Name of Ligand	PubChem ID	Interacting bonds
**1.**	Isoeugenitol	5318562	Thr199(2.86Å), Leu287(2.19 Å) and Leu272(4.8 Å)
**2.**	Syzyginin B	102445430	Glu166(2.82 Å), Leu141 (3.26 Å), Asn142 (2.48 Å), Gly143(2.64 Å), Glu166(2.82 Å), and Ser46(2.07 Å).
**3.**	Ellagic acid di-hexoside	5318135	Tyr237(2.99 Å), Asn238(2.43 Å), Lys137(2.68 Å), Asp197(2.93 Å), Thr199(2.19 Å), Arg131(2.93 Å), Asp289(3.17 Å), Leu287(3.34Å) and Met276(2.07 Å)
**4.**	Nobotanin D	14429414	Lys137(2.70 Å), Arg131(2.10 Å), Asp289 (2.81 Å), Thr199(2.58 Å) and Leu287(2.06 Å)
**5.**	Punicalgin	16129869	LYS137(2.44 Å), Arg131(2.70 Å), Asp197(2.49 Å), Gly195(3.02 Å), Asn133(2.74 Å), Asn238(2.18 Å), Thr199(2.85 Å) and Leu(2.02 Å)
**6.**	2 Hrptanone	8051	Asn221(2.11 Å)
**7.**	Eugenol	3314	Gln110(2.29 Å) and Phe294(3.79 Å)
**8.**	Eugenol Acetate	7136	Phe294(4.30 Å, 5.40 Å), Ile106(4.91 Å) and Val104(4.49 Å, 4.71 Å)
**9.**	2-methoxy-4-propylphenol	17739	Lys97(2.84 Å), Trp31(5.06 Å), Leu75(5.48 Å), Val73(4.25 Å) and Ala70 (4.32 Å).
**10.**	Eugenyl Benzoate	62362	Gln110(2.13 Å), Ile106(4.76 Å), Thr111(2.15 Å) and Phe294(4.88 Å)
**11.**	Vanilloloside	44577222	Met165(3.94 Å), His41(4.36 Å), His163(4.68 Å), Phe140 (3.62 Å), Ser144(2.99 Å) and Cys145(2.44Å)
12.	EugenylFormate		Gla110(2.76 Å) and Thr111(2.01 Å)
**13.**	DMPX	99562	Met165(3.51 Å), Gln (3.30 Å), His41(2.96 Å) and Cys145(3.06 Å)
**14.**	BS-DMPX	9843843	Gln189(3.30 Å), Met165(3.51 Å), His41(2.18 Å) and Cys145(3.08 Å)
**15.**	MS-DMPX	10760308	Lys137(2.04 Å), Arg131(2.81 Å), Asp197(3.13 Å), Tyr239(5.10 Å), Tyr237(3.56 Å), Leu286(5.20) and Let287(4.45 Å).
**16.**	Eugenyl-GX	5317538	Asn221(2.65 Å), Phe219(3.29 Å), Phe223(4.82 Å), Glu270(4.00 Å) and Asn227(3.47 Å)
**17.**	Syringin	5316860	Lys137(2.46 Å), Arg131(2.76 Å), Asp197(3.79 Å), Thr199(3.08 Å), Tyr239(5.20 Å), Leu287(5.30 Å) and Leu272(5.12 Å)
**18.**	Eugenol Glucoside	9945189	Cys85(5.21 Å, 4.58 Å) and Arg40(2.59 Å, 2.30 Å and 2.74 Å)
**19.**	Citrusin D	131752609	Phe140(3.35 Å), Glu166(4.78 Å), His163(2.61 Å) and Cys145(3.60 Å)
**20.**	Pentagalloylglucose	65238	Lys137(2.85 Å), Arg131(2.98 Å), Thr199(3.07 Å), Asn238(2.57 Å), Tyr237(5.04 Å), Lys236(3.98 Å), Leu272(3.96 Å), Ala285(3.38 Å) and Leu287(3.08 Å)
**21.**	Eugeniin	442679	Asp197(2.15 Å), Lys137(2.31 Å), Arg131(2.31 Å), Thr199 (2.92 Å), Glu290(3.20), Glu288(3.32 Å), Ley287(2.02 Å), Leu286(3.40 Å), Met 276(2.81 Å) and Gly275(3.35 Å)
**22.**	CHEMBL1076705	44242636	Thr199(3.02 Å), Asp197 (3.79 Å), Arg131 (2.76 Å), Lys137 (2.46Å) Leu287 (3.53 Å), Leu287(5.30 Å), Leu272(5.12 Å) and Tyr239(5.20 Å)
**23.**	2 Heptyl Benzoate	243678	Lys137(2.21 Å), Arg1312.26 Å), Asp289(3.21 Å)

			andLeu2874.13 Å)
**24.**	Biflorin	441959	Leu271(3.46 Å) and Leu287(2.51 Å, 3.95 Å and 5.12)
**25.**	Protopine	4970	Leu287(2.51 Å, 3.95 Å and 5.12) and Leu271(3.46 Å)
**26.**	Digalloylglucose	129628549	Tyr239(5.95 Å), Leu287(2.23 Å), Thr199(2.19 Å), Asp289(3.17 Å), Thr198(3.34 Å), Arg131(3.16 Å), Lys137(2.22 Å), Asp197(3.16 Å) and Asn133(2.06 Å)
**27.**	2-[(Galloyloxy)Methyl]-Alpha-D-Ribofuranose 5-(3,4,5-Trihydroxybenzoate)	9982777	Thr24(2.94 Å), Ser46(2.10 Å), His41(3.53 Å), Thr26(2.46 Å), His41(3.53), Glu166(2.10 Å), and ser144(2.27 Å)
**28.**	Hamamelitannin	44584241	Phe140(3.30 Å), His163(2.00 Å), Leu141(3.11 Å), Ser144(2.91 Å), G;y143(2.25 Å), Thr26(2.13 Å), Thr25(2.40 Å) and Thr45(2.90 Å)
**29.**	1,6- Digalloylglucose	118431216	Thr135(2.51 Å), Asn133(2.11 Å), Val171(5.01 Å), Asp197(3.60 Å), Thr199(3.09 Å) and Leu287(4.76 Å)
**30.**	2, 6 –Digalloylglucose	14034261	Cys85(5.16 Å), Phe181(2.08 Å), Phe185(3.36 Å), Arg188(2.82 Å), and Tyr54(2.91 Å)
**31.**	1,2-Digalloylglucose	54087871	Lys137(2.28Å), Asp197(4.05Å), Asn238(2.99Å), Asp289(3.40), Thr198(2.28Å) and Thr199(1.75Å).
**32.**	1,6-Bis-O-Galloyl-Beta-D-Glucose;	440221	Phe181(2.44Å), Cys85(3.65Å), Arg40(2.25), Glu55(2.51Å), Tyr54(2.87Å) and Arg188(2.60Å)
**33.**	(2S)-nonan-2-ol	11094758	Lys5(4.66Å), Phe291(5.34Å), Trp207(4.47Å) and Leu282(5.10Å)
**34.**	Nonan-2R-Ol	11286536	Trp218(5.26Å) and Leu271(4.38Å)
**35.**	Acetyleugenol	7136	Ser158(3.55 Å), Asn151(2.25 Å) and Gln110(2.18 Å)
**36.**	1-Methyloctyl Acetate	85788	Tyr237(4.74 Å), Leu272(4.66 Å), Leu287(4.07 Å) and Met276(4.10 Å).
**37.**	2-Heptanone	8051	Phe294(4.54 Å, 4.98 Å) and Asn151(2.44 Å)
**38.**	[(3S)-octan-3-yl] butanoate	76959495	Arg279(2.50 Å) and Gly275(3.64 Å)
**39.**	[(3R)-octan-3-yl] butanoate	12779846	Lys137(2.09 Å), Thr199(2.60 Å), Leu287(3.02 Å, 2.55 Å, 5.04 Å) , Asp197(2.95 Å),and Asn133(2.42 Å)
**40.**	Crategolic Acid	3694932	Ile106(5.03 Å), Val104(5.21 Å), Phe294(4.39 Å), Pro293(4.46 Å), Val297(4.47 Å), Pro252(5.17 Å) and Ile249(4.50 Å)
**41.**	6,10,10-Trimethyl-2-Methylenebicyclo(7.2.0)Undec-5-En-3-Ol	6437586	Ile106(5.30 Å), Val104(4.35 Å), Phe294(4.54 Å), Pro293 (4.03 Å) and Ile249(4.54 Å)
**42.**	1,3,6-Tri-O-Galloyl-B-D-Glucopyranose	452707	Tyr237(3.27 Å), Met276(5.50 Å), Leu287(3.33 Å), Leu286(5.31 Å), Tyr239(3.27 Å), Thr199(2.90 Å), Asp289(3.43 Å) and Arg131(2.61 Å)
**43.**	Beta Carotene	5280489	Tyr239(5.03 Å), Tyr237(4.97 Å), Leu272(4.78 Å), Met276(4.16 Å), Leu2874(4.18 Å), Glu290(3.79), Lys137(1.82 Å), Asp289(3.21 Å) and Arg131(2.56 Å),
**44.**	1-Decanol	8174	Phe294(4.57 Å)
**45.**	Friedlein	244297	Ile106(5.31 Å), Val104(4.17 Å), Phe294(5.25 Å and 4.01 Å)
**46**	4-Epi-Friedelin	15559350	Leu286(5.14 Å), Leu287(5.13 Å) and Gly275(3.56 Å)
**47.**	Galactiol	11850	Asp295(3.09 Å), Thr111(2.25 Å) and Gln110(1.80 Å)
**48.**	Hetastarch	24846132	Leu287(3.78 Å), Tyr239(3.25 Å), Thr199(2.92 Å),

			Asp289(3.28 Å), Glu290(3.25 Å), Lys137(3.16 Å), Lys5(2.17 Å) and Gln127(3.09 Å).
**49.**	Hentriacontane	12410	Val104(3.91 Å and 4.01 Å), Phe294(4.46 Å and 4.01 Å) and Ile106(5.37 Å)
**50.**	Ascorbic Acid	54670067	Thr199(2.92 Å), Asp289(3.29 Å), Arg131(2.42 Å) and Lys137 (2.70 Å)
**51.**	6-O-Stearoyl-L-Ascorbic Acid	98045018	Met276(4.10 Å), Leu286(4.83 Å), Leu287(4.80 Å), Leu272(5.26 Å), Tyr(5.02 Å), Arg131(2.49 Å), Lys137(2.92 Å) and Thr199(2.11 Å)
**52**	Syringoside	5316860	Leu286(5.29 Å), Leu287(3.81 Å), Asp289(3.05 Å), Arg131(2.67 Å), Thr199(2.87 Å), Tyr239(5.26 Å) and Tyr237(3.49 Å)
**53.**	Oryzarol	58114970	Thr199(2.30 Å), Tyr237(4.79 Å), Leu272(5.13 Å), Leu287(4.85 Å) and Leu286(5.31 Å and 5.36 Å)

Supplementary Figure S20: The complex molecule of Syzyginin B against the main protease of RMSF plot of protein and ligand (a & b), c) protein ligands contacts timeline, d) protein secondary structure elements (SSE).

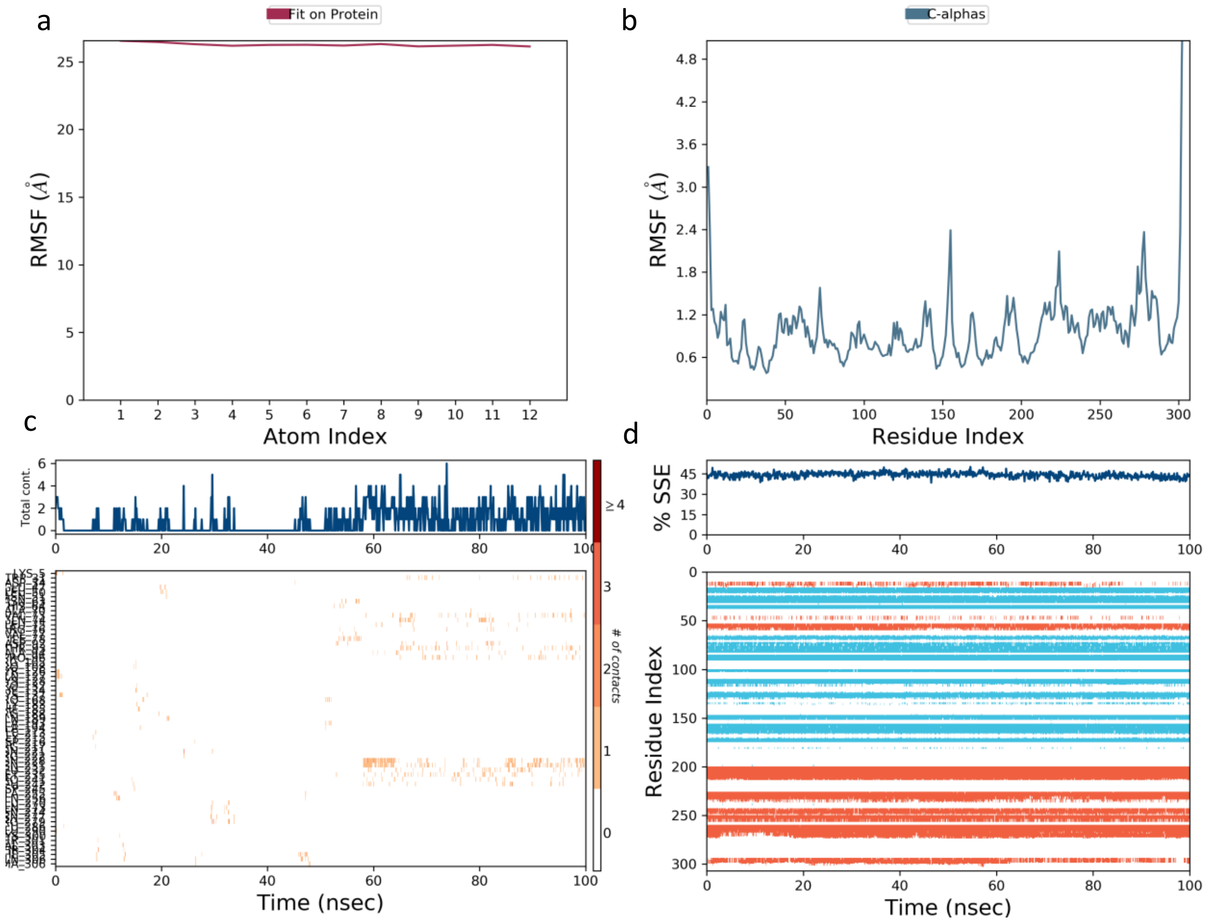

Supplementary Figure S21: The complex molecule of Eugeniin against the main protease of RMSF plot of protein and ligand (a & b), c) protein ligands contacts, d) protein secondary structure elements (SSE).

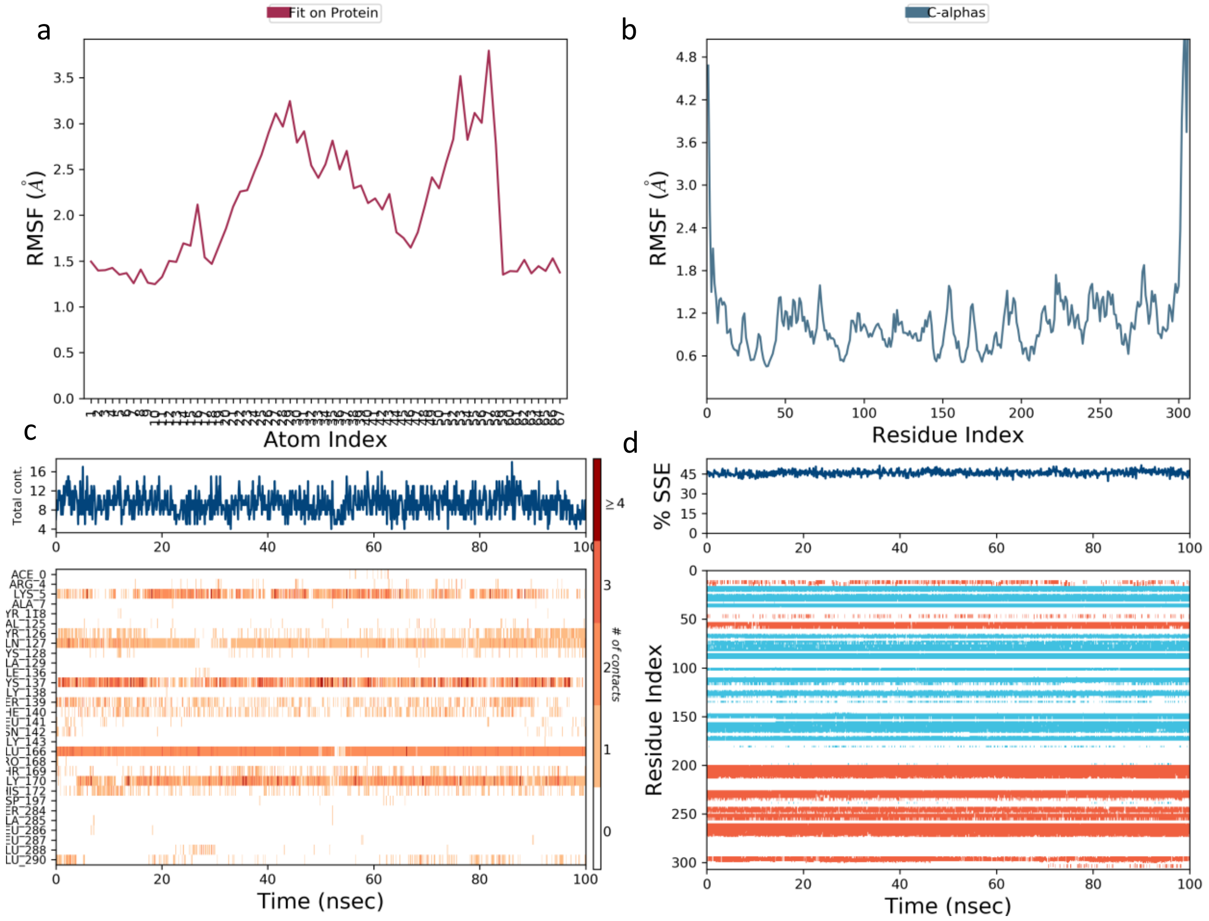

Supplementary Figure S22: The complex molecule of Eugenol against the main protease of RMSF plot of protein and ligand (a & b), c) protein ligands contacts timeline, d) protein secondary structure elements (SSE).

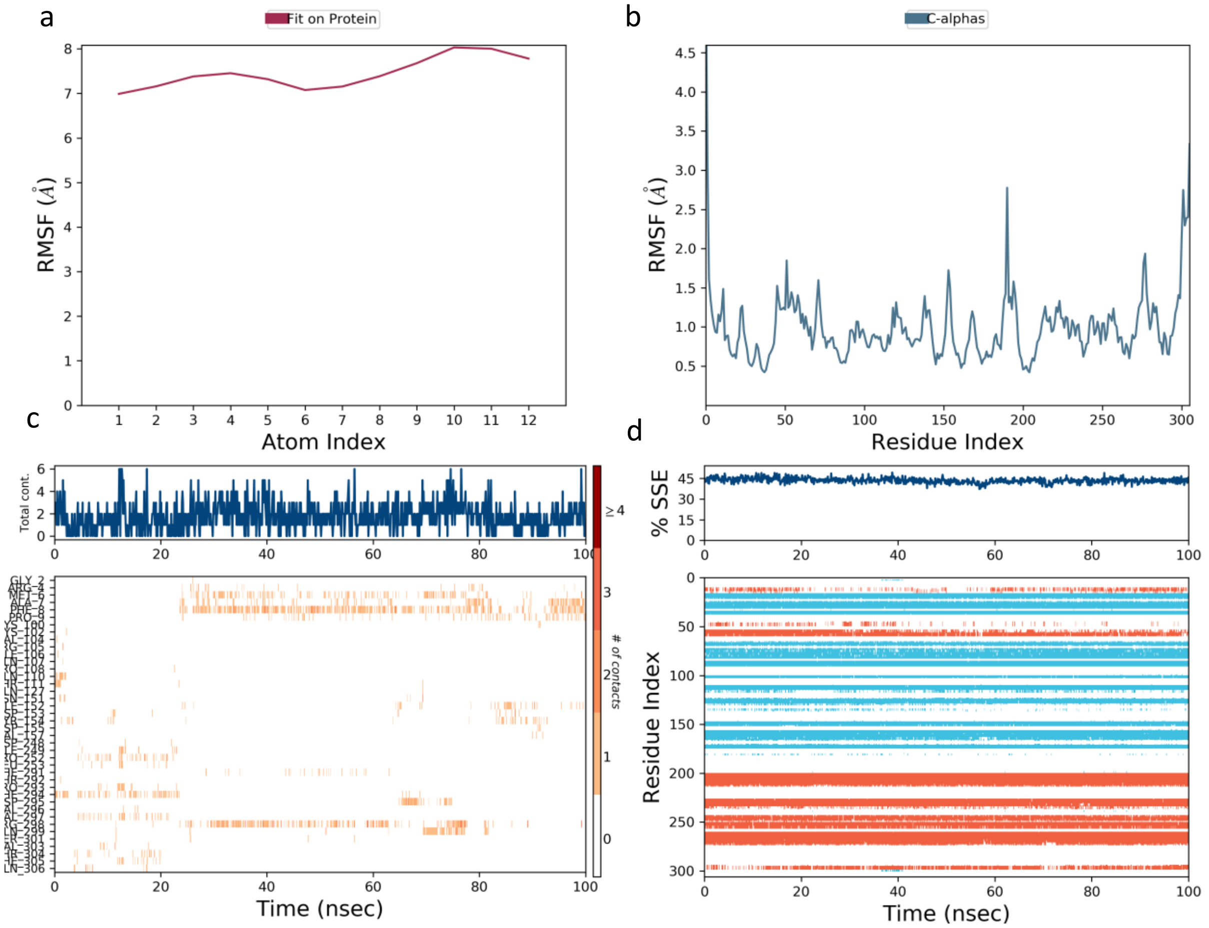

a
Fit on Protein
RMSF (Å)
Atom Index
b
C-alphas
RMSF (Å)
Residue Index
c
Total cont.
GLY_2
Time (nsec)
≥4
# of contacts
d
% SSE
Residue Index
Time (nsec)
